# Insights Into Platelet-Derived MicroRNAs in Cardiovascular and Oncologic Diseases: Potential Predictor and Therapeutic Target

**DOI:** 10.3389/fcvm.2022.879351

**Published:** 2022-06-09

**Authors:** Qianru Leng, Jie Ding, Meiyan Dai, Lei Liu, Qing Fang, Dao Wen Wang, Lujin Wu, Yan Wang

**Affiliations:** Division of Cardiology, Hubei Key Laboratory of Genetics and Molecular Mechanisms of Cardiological Disorders, Tongji Hospital, Tongji Medical College, Huazhong University of Science and Technology, Wuhan, China

**Keywords:** platelet, MicroRNAs, cardiovascular diseases, cancer, platelet-derived microvesicle (PMV)

## Abstract

Non-communicable diseases (NCDs), represented by cardiovascular diseases and cancer, have been the leading cause of death globally. Improvements in mortality from cardiovascular (CV) diseases (decrease of 14%/100,000, United States) or cancers (increase 7.5%/100,000, United States) seem unsatisfactory during the past two decades, and so the search for innovative and accurate biomarkers of early diagnosis and prevention, and novel treatment strategies is a valuable clinical and economic endeavor. Both tumors and cardiovascular system are rich in angiological systems that maintain material exchange, signal transduction and distant regulation. This pattern determines that they are strongly influenced by circulating substances, such as glycolipid metabolism, inflammatory homeostasis and cyclic non-coding RNA and so forth. Platelets, a group of small anucleated cells, inherit many mature proteins, mRNAs, and non-coding RNAs from their parent megakaryocytes during gradual formation and manifest important roles in inflammation, angiogenesis, atherosclerosis, stroke, myocardial infarction, diabetes, cancer, and many other diseases apart from its classical function in hemostasis. MicroRNAs (miRNAs) are a class of non-coding RNAs containing ∼22 nucleotides that participate in many key cellular processes by pairing with mRNAs at partially complementary binding sites for post-transcriptional regulation of gene expression. Platelets contain fully functional miRNA processors in their microvesicles and are able to transport their miRNAs to neighboring cells and regulate their gene expression. Therefore, the importance of platelet-derived miRNAs for the human health is of increasing interest. Here, we will elaborate systematically the roles of platelet-derived miRNAs in cardiovascular disease and cancer in the hope of providing clinicians with new ideas for early diagnosis and therapeutic strategies.

## Introduction

The latest WHO report released in December 2020 shows that non-communicable diseases (NCDs), represented by cardiovascular and oncologic diseases, have been the leading cause of global death over the past 20 years (1). Cardiovascular diseases (CVDs), principally ischemic heart disease, heart failure and stroke, account for one-third of the annual deaths and also are the major contributors to disability (2). However, the global incidence and mortality of CVDs have continued to rise uncontrollably, with an increase of 93 and 54%, respectively, for the last three decades (1, 3). The second cause of death is cancer, with 24.5 million incident cases worldwide and 9.6 million deaths in 2017 (4, 5). With the aging of social population, the deterioration of environment, the prevalence of obesity and the deterioration of lifestyle (smoking, alcohol, drugs nightlife, and physical inactivity), the incidence of cardiovascular and oncologic diseases is bound to grow. Despite massive researches funding and government spending, the ultimate efficacy of both diseases is far from satisfactory, most likely because of limited access to timely diagnosis and standard treatment. In fact, 5-year survival rates for heart failure and lung cancer are less than 25 and 17%, respectively (6, 7).

Platelets are small anuclear cell fragments in the circulating blood. Although platelets could not transcribe the gene, the complete translational and post-transcriptional regulation machinery including mRNA, non-coding RNAs, ribosomes, and initiation/termination factors are inherited from megakaryocyte and stored in the cytoplasm and granules during thrombopoiesis (8, 9). Platelet activation dependent on specific receptors [such as glycoprotein Ib-IX-V (GPIb-IX-V), purinergic receptors (P2Y1 and P2Y12), and integrin αIIbβ3] on their surface is the key step for platelet function (10, 11). Once activated, platelets quickly secrete granules and intracellular active substances such as P-selectin, soluble CD40 ligand (sCD40L), platelet factor 4 (PF4) and interleukin-1 beta (IL-1β) *via* exocytosis pathway. These substances contribute to platelet adhesion, aggregation, platelet-leukocyte crosstalk, platelet-endothelial crosstalk and systemic inflammation states (12–14). These actions are important mechanisms by which platelets are involved in thrombosis, cardiac remodeling after myocardial infarction, atherosclerosis, diabetic microangiopathy, tumor growth, and metastasis (13, 15–18). Besides these proteins, platelets inherit a variety of nucleic acids including non-coding RNAs (miRNAs as well as lncRNAs) and messenger RNAs (mRNAs). In recent years, in-depth transcriptional analyses identified up to 532 different miRNAs and as many as 3000–6000 mRNAs in human platelets (19, 20). As we known, miRNAs are highly conserved, small endogenous non-coding RNAs negatively regulating gene expression at the post-transcriptional level by complementary sequence recognition, and are involved in many pathophysiological processes, including cardiovascular diseases and cancer. An enormous amount of research has gradually described the strong biological effects of platelet-derived miRNAs. They not only regulate the synthesis of platelet protein but also are transferred to endothelial cells, smooth muscle cells (SMCs), macrophages, and tumor cells, where they bind to host cells’ mRNAs (21–23). However, a systematic review about the role and mechanism of platelet-derived miRNAs on two highly lethal diseases, cardiovascular disease and cancer, is lacking.

In this study, we explore the possibility of circulating platelet-derived miRNAs as early diagnosis and prognostic factors, and their roles in the occurrence and development of cardiovascular diseases and tumors, which may provide clinicians with new diagnostic and therapeutic targets for these diseases.

### The Origin of Platelets MicroRNAs

MicroRNAs are a class of regulatory non-coding RNAs with a length of ∼22 nucleotides expressed in multicellular organisms and synthesized by an elaborate system involving numerous protein-protein and protein-RNA interactions (24, 25). Briefly, miRNA-related genes are firstly transcribed into primary miRNAs (pri-miRNAs) by RNA polymerase II (Pol II), and subsequently pri-miRNAs are processed into shorter precursor miRNAs (pre-miRNAs) by a complex formed by the RNAase-III enzyme Drosha and its interaction partner DGCR8 in the nucleus. Next, pre-miRNAs are transported out of the nucleus by exportin-5. In the cytoplasm, RNase Dicer enzyme bound to the double-stranded RNA with protein TRBP cleaves pre-miRNAs into shorter double-stranded miRNAs. Finally, double-stranded miRNAs are replicated into argonaute 2 (Ago2) and form the miRNA-induced silencing complex (RISC). One strand of the double-stranded miRNA is retained in the RISC complex, and the other strand is expelled from the complex and rapidly degraded. The RISC complex containing the miRNA single strand can function in subsequent gene regulation processes (26).

Since platelets are anucleate, mature miRNAs in platelets were previously considered remnants of megakaryocytes. However, later studies confirmed that anucleate platelets possess complete elements (Dicer, Ago2, and TRBP2) which can machine precursor miRNA (pre-miRNA) into mature miRNA in their cytoplasm (27), but lack of nuclear microprocessor components Drosha and DGCR8. The function of platelet Dicer enzyme was confirmed since miRNA-sized RNA fragments were obtained when a radioactive Dicer substrate ^32^P-labeled pre-let-7a-3 was co-cultured with platelet extracts (27). Additionally, their research also confirmed that platelets harbor functional Ago2–miRNA complexes. The direct interaction between Ago2 with endogenous mature miR-223 was confirmed by northern blotting of platelets Ago2 immunoprecipitation and the inhibitory expression of target gene P2Y12 confirmed the regulatory function of the Ago2-miR-223 complex (27). These results suggested that platelets have the ability to produce mature miRNA by processing pre-miRNA templates.

**FIGURE 1:**
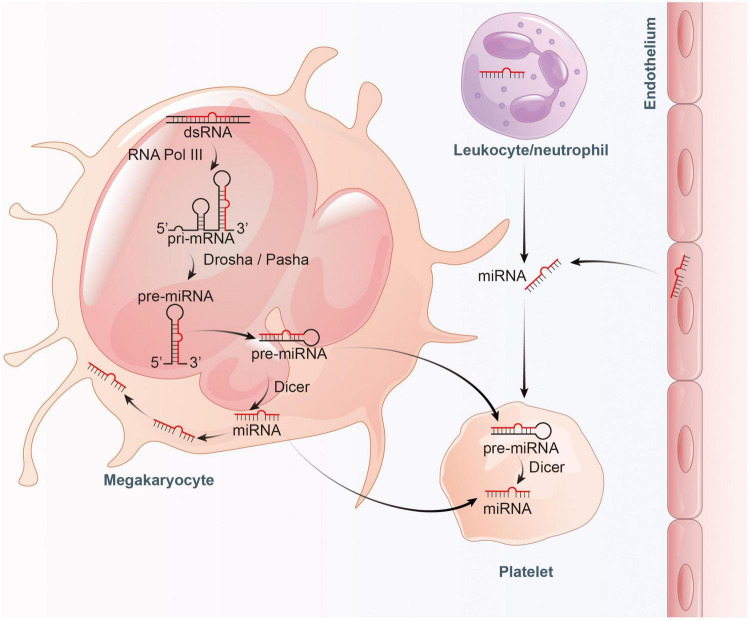
Proposed model for the inheritance and operation of platelet miRNAs. On the one hand, platelets inherit precursor miRNA (pre-miRNA) from megakaryocytes and process them into mature miRNA by Dicer. On the other hand, platelets also inherit mature miRNA from megakaryocytes, and absorb miRNA from endothelial cells and leukocytes.

However, evidence suggests that a large proportion of mature miRNAs contained in platelets are mainly inherited from megakaryocytes (28). Cultured megakaryocytes transcribes multiple miRNAs, which correlates well with the content of miRNAs found in platelets (28, 29). Platelets inherit RNA pool, including pre-miRNAs, from their parent megakaryocytes (30, 31). These RNAs become important sources of platelet miRNA as they may be processed into mature miRNA by functional Dicer as templates (22, 27). Besides, miRNAs can bidirectionally transfer between platelets and the surrounding environment (32). RNA uptake from contacting cells or the circulating blood is also thought to be a contributor to miRNA diversity within platelets (30, 33). In general, platelets can not only inherit a large amount of miRNAs from megakaryocytes and take up part of miRNAs from surrounding environment, but also can process the inherited and ingested RNAs into miRNAs (Figure 1).

### The Content of Platelets MicroRNAs

As early as in 2008, Bruchova et al. first detected abundant miR-26b in platelets in patients with polycythemia vera and essential thrombocythemia (34). This was the first study attracting our attention to platelet-derived miRNAs. Later, a large number of studies confirm that platelets contain a wide variety of miRNAs, although platelet-derived miRNAs are much less than those in nucleated blood cells (35, 36). With the development of microarray and sequencing technology, more miRNAs have been identified in platelets. Landry et al. identified 219 miRNAs in purified leukocyte-depleted platelet by locked nucleic acid (LNA) microarray profiling in 2009. Among them, miR-223, let-7c, and miR-19a were the three most abundant miRNAs according to their results (27). Subsequent studies on the most common miRNAs in platelets were inconsistent (Table 1). In many studies, miR-223-3p was described as the most abundant platelet-derived miRNA (37–39). Later, the first next-generation sequencing (NGS) data on platelet miRNAs was published, identifying 532 miRNAs present in platelets, with the let-7 family accounting for almost half of the total miRNA content (20). Other miRNA families highly represented in human platelets include miR-199, miR-103, miR-25, and miR-140. However, the expression of miR-223 was only ranked the tenth in this study. Overall, the top 15 miRNAs accounted for more than 90% of all miRNAs present in human platelets. More recently, Bray et al. analyzed miRNAs content in purified platelets from four healthy volunteers, expanding the number of known platelet miRNAs to approximately 750 (35). In their study, the top five miRNAs expressed most abundantly in platelets were miR-223, miR-451, miR-21, miR-23a, and miR-126. In Table 1, we summarized the top 20 miRNAs in platelet.

**TABLE 1:** A summary of the top 20 expressed miRNAs in platelets.

Total miRNA numbers in platelets	The top 20 miRNAs in platelets	Method	References
219	miR-142-5p, miR-142-3p, miR-223, let-7a, miR-185, let-7c, let-7i, let-7b, miR-126, miR-103, miR-320, miR-30c, miR-130a, miR-26a, miR-191, miR-30b, miR-146a, miR-23b, miR-21, miR-23a	Locked nucleic acid (LNA)-based microarray profiling in human platelets	(27)
532	Let-7f-5p, let-7a-5p, miR-199ab-3p, miR-103a-3p, miR-140-3p, miR-7g-5p, miR-25-3p, let7b-5p, miR-7d-5p, miR-21-5p, miR-185-5p, miR-191-5p, miR-223-3p, miR-26b-5p, miR-423-5p, miR-221-3p, miR-107, miR-101-3p, let-7i-5p, miR-23a-3p	High-throughput sequencing in purified human platelets	(20)
750	miR-223, miR-22, miR-21, miR-126, miR-23a, miR-451, miR-17, miR-191, miR-26b, miR-15b, miR-23b, miR-484, miR-19a, let-7g, miR-221, miR-130a, miR-425, miR-142-5p, miR-185, miR-30d	RNA sequencing in highly purified, leukocyte-depleted platelet	(35)

### MicroRNAs and Platelet Biogenesis

**FIGURE 2:**
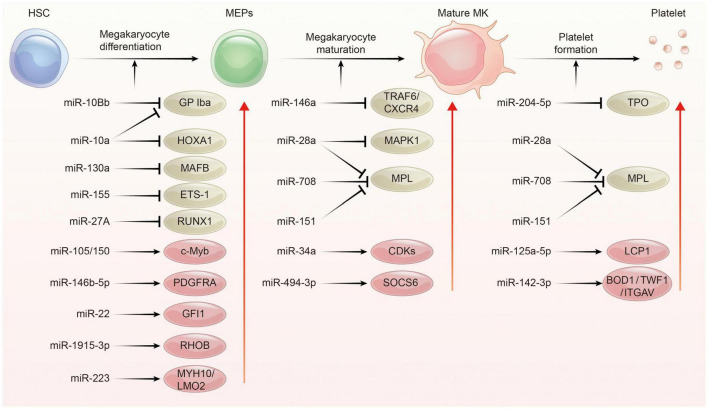
The regulation network of miRNA during platelet biogenesis. Platelet production goes through three main stages: Firstly, hematopoietic stem cells (HSCs) were differentiated into megakaryocyte erythroid progenitor cells (MEPs), which were further differentiated and proliferated to form mature megakaryocytes, and then produced functional platelets. This process is controlled by several miRNAs through regulating the expression of their target gene. The red circle represents miRNA-mRNA pairs promoting platelet biogenesis, and the brown color represents miRNA-mRNA pairs inhibiting this process. Abbreviations: GP Iba, glycoprotein Ib platelet subunit alpha; HOXA1, homeobox A1; MAFB, V-maf musculoaponeurotic fibrosarcoma oncogene homolog B; EST-1, E26 transformation-specific sequence 1; RUNX1, runt-related transcription factor 1; PDGFRA, platelet derived growth factor receptor alpha; GFI1, growth factor independent 1; RHOB, ras homolog family member B; MYH10, myosin heavy chain 10; LMO2, LIM-only protein 2; TRAF6, tumor necrosis factor receptor associated factor 6; CXCR4, C-X-C motif chemokine receptor 4; MAPK1, mitogen-activated protein kinase 1; MPL, thrombopoietin receptor; CDKs, cyclin dependent kinases; SOCS6, suppressor of cytokine signaling 6; TPO, thrombopoietin; LCP1, L-plastin; BOD1, biorientation of chromosomes in cell division 1; TWF1, twinfilin actin binding protein 1; ITGAV, integrin subunit alpha V.

Platelets are derived from megakaryocytes, and formed when the edges of mature megakaryocytes break off. Each megakaryocyte releases about 1,000–5,000 platelets (40, 41). The process of platelet production lasts approximately 7 days, involving in three main phases: megakaryocyte differentiation, megakaryocyte maturation, and platelet formation. This process is complex and regulated by multiple mechanisms including epigenetic, transcriptional as well as post-transcriptional gene expression control. Indeed, several studies have addressed miRNAs as well as their target proteins play important roles in megakaryocytopoiesis and platelet biogenesis (Figure 2; 21, 22, 29, 42).

In the megakaryopoiesis stage, hematopoietic stem cells (HSCs) differentiate and grow into megakaryocyte erythroid progenitor cells (MEPs) in bone marrow, which then develop to megakaryocytes under the influence of different factors such as thrombopoietin (TPO). Garzon et al. firstly explored the differential expression of miRNAs profiles between cultured CD34+ hematopoietic progenitor cells and megakaryocytes (43). They found a strong downregulation of 19 miRNAs during megakaryocytopoiesis suggesting that these downregulated miRNAs possibly unblock certain genes involving this process. For example, MAFB (v-maf musculoaponeurotic fibrosarcoma oncogene homolog B) and HOXA1 (Homeobox A1) gene are upregulated during megakaryocyte differentiation, as they are targets for the downregulated miR-130a and miR-10a, respectively (43). In contrast, miR-34a increases during megakaryocytic differentiation and stimulates megakaryocytopoiesis by enhancing megakaryocyte colony formation from CD34 + HSCs (44). Additionally, hematopoietic stem cells with miR-150 overexpression produced 8-fold enrichment of megakaryocyte *in vitro* and 15-fold amplification *in vivo* compared with normal controls. miR-150 was shown to maintain normal differentiation of MEPs into megakaryocyte by targeting the transcription factor c-myb (45). On the contrary, miR-28 attenuates TPO stimulating signal by downregulating the thrombopoietin receptors (TPOR, MPL), resulting in a negative effect on megakaryocyte differentiation (46). Recently, other miRNAs such as miR-10a, miR-155, and miR-125a-5p were also shown to play an important role in the formation of megakaryocytes (47–49). Interestingly, mature platelets release microvesicles (PMVs) containing many miRNAs can be internalized by bone marrow hematopoietic stem cells and regulate megakaryocytes biogenesis as a self feedback regulatory mechanism (50, 51). MiR-223, the most abundant miRNA in PMVs, enhances MK differentiation and maturation by inhibiting MYH10 and LMO2 (52). Additionally, global miR-223 knockout leads to an obstacle in the recovery of platelet production after platelet immunodepletion in mice, supporting the role of miR-223 in thrombopoiesis (53). Another miRNA, miR-1915-3p, which is highly enriched in PMV, exhibits more significant effects than miR-223 in promoting MK differentiation by suppressing Rho GTPase family member B (RHOB) expression (54).

In the thrombopoiesis stage, megakaryocytes greatly enlarge their bodies with amplifying their DNA to 64-fold, filling with a high concentration of ribosomes, and synthesizing lots of platelet-specific proteins (55–57). Then, an expansive and interconnected membranous network of pools and tubules is formed, also named the demarcation membrane system (DMS) (58). DMS divides the megakaryocyte cytoplasm into small chamber where pro-platelets are split out (59, 60). TPO is widely regarded as the primary regulator of thrombopoiesis for their promoting roles on megakaryocyte endomitosis by binding to the c-Mpl receptor (61, 62). MiR-204-5p and miR-28a were reported to directly target TPO and MPL, respectively, *via* sequence-dependent 3′-UTR repression and inhibit platelet formation (46). Additionally, miR-142-3p was reported to maintain actin filament homeostasis, thereby promoting actin-dependent pro-platelet formation (63). miR-125a-5p directly targets and reduces the expression of L-plastin, an actin-bundling protein who inhibits the pro-platelet formation (49). Other miRNAs, such as miR-125b and miR-660 were found to promote platelet output from cultured megakaryocytes while miR-23a/27a/24-2 cluster blocked this process (64).

### Platelet MicroRNAs and Platelet Activation

Platelets are unstable and keep hyperresponsiveness to external stimuli, such as endothelial injury, infection, and metabolic disorders. Platelet receptors, such as glycoprotein (GPIb and GPVI), adenosine receptors (P2Y12 and P2Y1), thromboxane a2 receptor (TP), and thrombin receptors (PAR1, PAR3, and PAR4), act as switches for platelet activation once binding to their ligands von Willebrand factor (vWF), collagen, ADP, TxA2, and thrombin (65, 66). MiRNAs, as an important part of post-transcriptional regulation of platelet proteins, regulate platelet activity by directly targeting several platelet proteins (Table 2). Landry et al. firstly reported that Ago2⋅miR-223 complexes negatively regulated the expression of P2Y12 receptor by targeting the 3′-UTR region (27). However, the results of studies assessing the effect of miR-223 on platelet activation are contradictory. Leierseder et al. found that miR-223 did not affect platelet activation and aggregation and bleeding time while Elgheznawy et al. reported that miR-223 deletion in mice exacerbated platelet aggregation and the formation of large thrombosis (53, 67). Additionally, miR-126 transfection inhibits platelet reactivity by downregulating the expression of a disintegrin and metalloproteinase-9 (ADAM9), a protease associated with the interaction between platelet adhesion and collagen, and P2Y12 receptor expression (68, 69). In a LPS-induced sepsis model, platelet miR-26b is significantly downregulated, which contributes to elevated P-selectin (SELP) expression of MKs and platelets, and augments platelet activation (70). MiR-181a targets Ras-related protein 1B (RAP1B), an important protein participating in platelet activation and hemostasis induced by agonists, and thereby reducing platelet activation (71). Recently, high-through RNA sequencing was used to compare miRNA and mRNA profiles between hypo- and hyper-reactive platelets and helped to discover more miRNA-mRNA pairs associated with platelet activation. Nagalla et al. reported that 74 miRNAs were significantly changed in epinephrine-activated platelets compared with resting subjects. Several changed miRNAs were negatively correlated with the genes related with platelet activation, such as miR-200b: PRKAR2B, miR-495: KLHL5, and miR-107: CLOCK. The regulatory relationship was validated by miRNA-mediated inhibition of the targeted genes. Moreover, the function of these miRNA-mRNA pairs was further confirmed by reduced activation in platelets lacking PRKAR2B (72).

**TABLE 2:** Overview of key platelet miRNAs and their function within platelets.

MicroRNA	Target	Role in platelets	Study samples	References
miR-223	P2y12R	Platelet activation	Mice/MEG-01	(158, 144)
	SMOC1	Platelet activation	Mice	(230)
	Factor XIII	Platelet activation	Mice	(67)
miR-126	P2y12R	Platelet activation	Mice	(146)
	PLXNB2	Platelet activation	Human	(68)
	ADAM9	Platelet aggregation	Mice	(69)
miR-30c	PAI-1	Thrombus formation	Mice	(149)
miR-148a	FcγRIIA	Thrombus formation	Mice	(231)
miR-181a	RAP1B	Platelet activation	Mice	(71)
miR-320c	RAP1	Platelet activation	Human	(232)
miR-140	SELP	Platelet activation	MEG-01 MK	(144)
miR-26b	SELP	Platelet activation	MEG-01 MK	(144)
miR-200b	PRKAR2B	Platelet reactivity	MEG-01 MK	(72)
miR-495	KLHL5	Platelet reactivity	MEG-01 MK	(72)
miR-107	CLOCK	Platelet reactivity	MEG-01 MK	(72)
miR-96	VAMP8	Platelet secretion	Human	(233)
miR-376c	PCTP	Platelet secretion	Human	(84)
miR-21	WASp	TGF-β1 secretion	Mice	(234)
miR-236	Bcl-xl/Bak1	Platelet *apoptosis*	Human	(235)

*SMOC1, secreted modular calcium-binding protein 1; PLXNB2, plexin B2; ADAM9, metalloproteinase domain-containing protein 9; PAI-1, plasminogen activator inhibitor-1; RAP1B, ras-related protein 1b; SELP, selectin P; PRKAR2B, cAMP-dependent protein kinase type II-beta regulatory subunit beta; KLHL5, Kelch like family member 5; VAMP8, vesicle associated membrane protein 8; PCTP, phosphatidylcholine transfer protein; WASp: Wiskott–Aldrich syndrome protein.*

### Platelet MicroRNAs and Platelet Secretion

Platelets contain three types of secretory organelles—alpha and dense granules and lysosomes. Alpha granules are the largest and most abundant secretory granules in platelets. More than 280 proteins are stored in alpha particles, including vWF, PF4, P-selectin, and platelet-derived growth factor (PDGF) (73, 74). Dense granules contain more than 200 small molecules, mainly including calcium, ADP/ATP, and 5-hydroxytryptamine (75). Lysosomes only account for a small proportion in platelet and have more heterogeneous in composition and properties, containing a number of acid hydrolases, cathepsins, and lysosomal membrane proteins (76). In addition, a large number of miRNAs may also be stored in these granules, since the circulating platelet-derived miRNA increases significantly, when these particles are secreted after platelet activation (36). However, what we still don’t know is their distribution and content in these particles.

The release of platelet granules is the material basis of platelet functional diversity and involves in the occurrence and development of many diseases especially cardiovascular and oncologic diseases (77). These platelet granules can fuse to the membrane of platelets *via* complex mechanisms after platelet activation and release their contents into the extracellular vascular space (78–82). The molecular mechanisms about platelet secretion mainly involve in soluble *N*-ethylmaleimidesensitive factor attachment protein receptor (SNARE) families (83). In 2010, Kondkar et al. reported that miR-96 overexpression resulted in significant inhibition of the expression of vesicle-associated membrane protein 8 (VAMP8), a SNARE protein which was elevated in hyperactive platelets and crucial for promoting platelet granules secretion (Table 2). In addition, miR-376c was found to inhibit the expression of phosphatidylcholine transfer protein (PCTP), an important protein contributing to secretion of dense granules by regulating PAR4-mediated platelet activation (84). Notably, the above-mentioned miRNAs who are involved in regulating platelet activation may also alter platelet particles secretion theoretically, since platelet exocytosis are initiated by platelet activation.

### Platelet Secretes and Delivers MicroRNAs

In earlier studies, researchers found that the changes of circulating miRNA were closely related to platelets activation. They identified a large number of miRNAs when comparing the miRNAs profiles in hyporeactive platelets and hyperreactive ones in response to agonist stimulation (72). However, what puzzles scientists is how they are released and maintain their stability after entering into circulation? In the past, the concept that platelets produce many membrane encapsulated extracellular vesicles (EVs) has been widely accepted (74, 85). These EVs hide miRNAs in their natural membrane barrier and isolate them from degradative components (such as nucleases) in the extracellular environment, maintaining the stability of extracellular miRNAs. Later, researchers demonstrated that platelet secreted miRNAs into circulation possibly through EVs-mediated manner (86, 87). They compared miRNAs profiles between plasma microparticles (MP) and MP-free plasma and confirmed that plasma miRNAs mainly originated from microparticles. 41–45% of circulating microparticles were of platelet, 28% of leukocyte, and 8% of endothelial origin.

**FIGURE 3:**
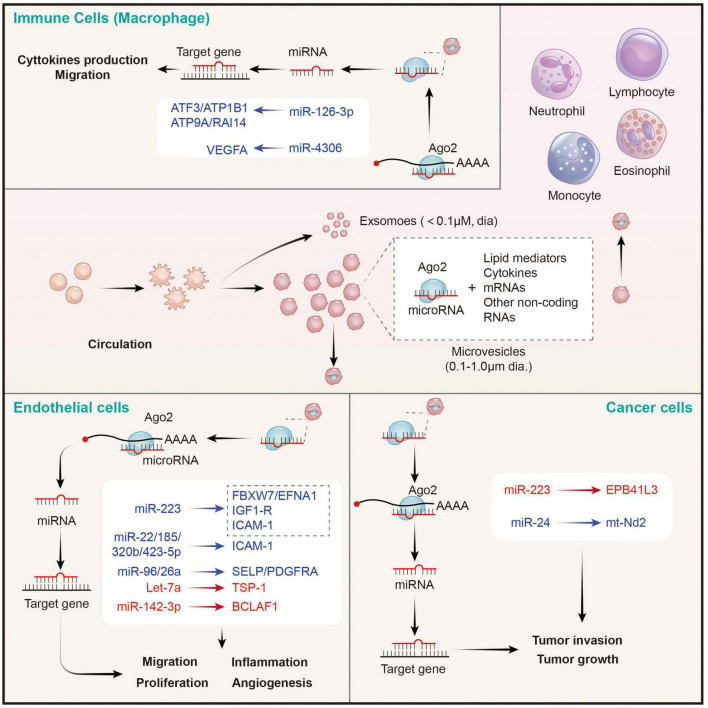
Platelet MPs mediate the transfer of intercellular miRNAs to other cells in the circulatory system, and participate in the regulation of gene expression of recipient cells. MPs released by activated platelets are rich in bioactive lipid mediators, cytokines, mRNAs and a wide variety of non-coding RNAs (including miRNAs). Platelets derived miRNAs can be efficiently transferred into endothelial cells, immune cells, and cancer cells through MPs-mediated manner. Platelet MPs form a tent to protect miRNAs from extracellular nucleases degradation and act as intercellular transporters to deliver functional Ago2⋅microRNA complexes, through which they modulate the genes of recipient cells in the circulatory system and perform a wide range of biological functions. The red represents miRNA-mRNA pairs that produce promoting functions and the blue represents miRNA-mRNA pairs that produce inhibitory functions. Abbreviations: Ago2, argonaute 2; ATF3, activating transcription factor 3; ATP1B1, sodium/potassium-transporting ATPase subunit beta-1; ATP9A, ATPase phospholipid transporting 9A; RAI14, retinoic acid induced 14; VEGFA, vascular endothelial growth factor A; FBXW7, F-box and WD-40 domain protein 7; EFNA1, ephrin A1; IGF1-R, insulin like growth factor 1 receptor; ICAM-1, intercellular adhesion molecule 1; SELP, selectin P; PDGFRA, platelet derived growth factor receptor alpha; TSP-1, thrombospondin 1; BCLAF1, BCL2 associated transcription factor 1; EPB41L3, erythrocyte membrane protein band 4.1 like 3; mt-Nd2, mitochondrially encoded NADH: ubiquinone oxidoreductase core subunit 2.

Upon stimulation, platelets mainly secrete two types of EVs: exosomes (derived from exocytosis of multivesicular bodies and alpha-granules, <100 nm) and microvesicles (produced by surface shedding, 100 nm–1 μm) (74). Although exosomes from endothelial cells and tumor cells have been verified to be carriers for miRNA-based intercellular communication and a source of circulating miRNAs (88), the role of platelet-derived exosomes in miRNA transfer is still undetected. Philipp Diehl et al. firstly proposed that circulating miRNAs were mainly localized in microvesicles (MVs) derived from different tissues, especially from platelets (87). Consistently, Laffont et al. reported that platelet-derived microvesicles (PMVs) acted as intercellular transporters delivering rich miR-223 to endothelial cells and altering the gene expression (86). Thus, the transfer of platelet miRNAs is mainly mediated by PMVs (89). Indeed, platelet-derived MVs are the major source of cell-derived MVs in the circulation (90), which is consistent with the conclusion that circulating miRNAs are mainly platelets-derived (91). Then, we elaborate on the regulation of PMVs mediated miRNAs on distant cells below (Figure 3).

#### Platelet Microvesicles Transfer MicroRNAs to Endothelial Cells

Takeuchi et al. found that platelet-like particles (PLPs) derived from the megakaryoblastic cell line Meg-01 transferred labeled RNA to the endothelial recipient cells. This is the first evidence supported the phenomenon of platelet-mediated miRNA transfer (92). Later, Laffont et al. found that endothelial cells actively uptake PMVs produced by activated platelets, resulting in a significant increase of platelet miRNA (such as miRNA-223) in HUVECs when they are co-incubated. Moreover, miR-223-Ago complexes in PMVs was found to have regulatory ability, resulting in significant downregulation of the expression of two endogenous target genes FBXW7 and EFNA1 (86). Consistently, increased miR-223 in thrombopoietin-induced platelets leads to decreased IGF1-R expression in cultured endothelial cells, and exacerbates their apoptosis (93). Additionally, platelet-derived miR-223 also was predicted to target the ICAM-1 gene and proved to inhibit its expression in HUVECs during inflammation process *via* blocking the NF-κB and MAPK pathways (94). Besides miR-223, co-culture of fluorescent labeling platelets with miR-Scr-FITC and HMEC-1 endothelial cells confirms that 4 platelet-derived miRNAs (miR-22, −185, −320b, and −423-5p) are uptake by endothelial cells and restrains the expression of ICAM-1 (23). Moreover, miR-96 and −26a were also found to be transferred from PMVs to HUVECs, and inhibited the migration and tube formation of HUVECs (95). In conclusion, platelet-derived miRNAs are taken up by endothelial cells through microvesicles, inhibit endothelial inflammation, migration and tubule formation, and promote apoptosis. However, the opposite results also exist. Let-7a highly expressed in PMVs was found to significantly promote endothelial cell angiogenesis by targeting the anti-angiogenic molecule thrombospondin-1 (TSP-1) (96). Moreover, miR-142-3p from PMVs was also found to be delivered into endothelial cells and enhanced their proliferation *via* inhibiting the expression of Bcl-2-associated transcription factor (BCLAF)1 (97). Therefore, the effects of platelet-derived miRNAs on endothelial cells depend on the type of miRNA ingested. However, it is still unclear whether the uptake of platelet-derived miRNA types by endothelial cells is selective in different states, or only depends on the content of miRNAs in PMVs. It is worth noting that SMCs are identified as other recipients on vascular for PMV-mediated miRNAs. Thrombin-stimulated platelets produced numerous PMVs containing miR-223, miR-339, and miR-21, which were transferred into SMCs and inhibited their proliferation by downregulating the expression of platelet-derived growth factor receptor beta (PDGFRb) (98).

#### Platelet Microvesicles Transfer MicroRNAs to Immune Cells

Circulating leukocytes are a kind of cells that can be directly coupled with activated platelets through surface receptors, and subsequently modifying their phenotypes. In this study, we focused on leukocytes as recipient cells that receives miRNAs from PMVs and the biological effects of the miRNAs on the former. Laffont et al. found that fluorescently labeled PMVs were internalized by primary human macrophages when they were co-incubated, subsequently causing the aggregation of miR-126-3p in the recipient macrophage (99). Further analysis using transcriptome-wide microarray, 34 miRNAs were identified to be significantly elevated in macrophages upon incubation with PMVs. Correspondingly, 367 mRNAs, including important cytokines and chemokines such as CCL4, CSF1, and TNF-α, acting as potential targets for these changed miRNAs, were confirmed to be significantly downregulated. Co-incubation of macrophages with PMVs enhanced their ability of phagocytosis, pointing toward a potential role of PMVs-mediated miRNAs in shaping macrophage functions (99). However, it didn’t identify which miRNAs besides miR-126-3p are involved in the cellular reprogramming of macrophages. A later study from Yang et al. suggested that miRNA-4306 mainly from PMVs was effectively delivered into human monocyte-derived macrophages, and inhibited their migration *in vitro* and reduced macrophage infiltration in myocardial infarction tissues. The inhibitory effect of miR-4306 on macrophages was possibly mediated by restraining the ERK/NF-κB signaling (100). Besides macrophages, PMVs also interact with natural killer (NK) cells and shift their function *via* transferring platelet-derived miR-183 and suppressing the expression of NK activation adapter DAP12 (101). However, the mechanisms of PMV-miRNAs transfer and mediated gene regulation on other immune cells require further exploration.

#### Platelet Microvesicles Transfer MicroRNAs to Cancer Cells

Although the role of PMVs in tumor regulation has been largely confirmed, there are few studies on whether the miRNAs carried by PMVs have direct effects on tumor cells (102). In 2015, Liang et al. found that PMVs-derived miR-223 was rapidly delivered into human lung cancer A549 cells once they were nurtured together. Subsequently, the invasion ability of A549 cell was increased. This effect may be explained by the inhibition of tumor suppressor-associated gene EPB41L3 by platelet miR-223 in A549 cells (103). By contrary, Michael et al. reported that platelet-derived RNAs, including miRNAs, were transferred into tumor cells, leading to tumor cell apoptosis as their vector PMVs left the circulation and entered the tumor microenvironment. MiR-24 was a major species in this transfer (104). These findings provide novel insights of horizontal miRNA transfer from PMVs to tumor cells and their roles on cancer progression. However, the mechanisms of PMVs infiltration and miRNA transfer, as well as the types of transferred miRNAs and global effects on tumor gene expression, remain to be further investigated.

### Functions of Platelet-Derived MicroRNAs in Cardiovascular Diseases and Cancer

**FIGURE 4:**
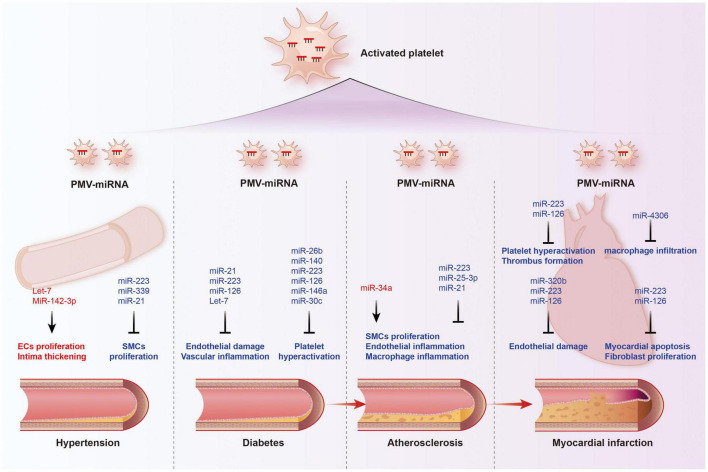
Functions of platelet-derived miRNAs in cardiovascular diseases. The transport of miRNAs from platelet microvesicle (PMV) to the cardiovascular system participate in the occurrence and development of hypertension, diabetes, coronary heart disease, and myocardial infarction, involving multiple mechanisms such as endothelial homeostasis, smooth muscle cell proliferation, inflammatory cell infiltration, and cardiomyocyte apoptosis. Red labeled miRNAs promoted their downstream processes, while blue labeled miRNAs inhibited the downstream processes.

**FIGURE 5:**
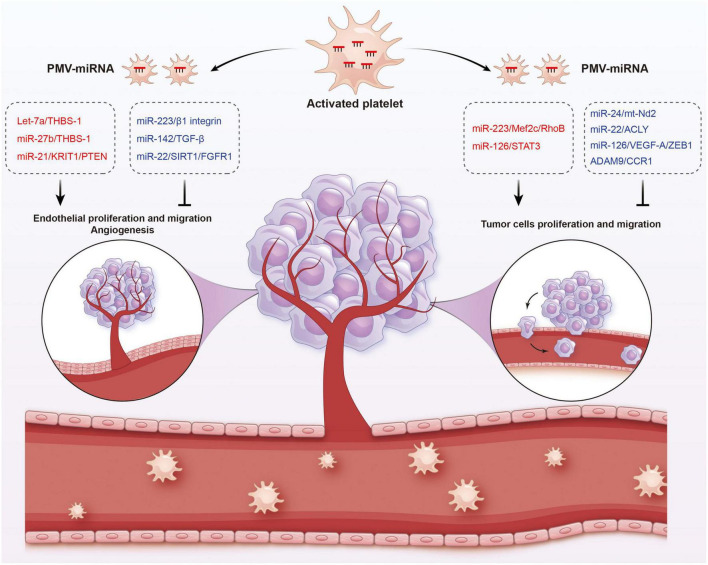
Functions of platelet-derived miRNAs in cancer. Platelet-derived miRNAs change tumor fates in two ways: on the one hand, PMVs transfer platelet miRNAs to vascular endothelial cells and are associated with enhanced tumor metastasis and cancer progression; on the other hand, PMVs can penetrate the blood vessels and enter the tumor microenvironment to directly transfer platelet miRNAs into tumor cells, thus regulating gene expression in tumor cells and tumor progression. The red represents miRNA-mRNA pairs that produce promoting functions and the blue represents miRNA-mRNA pairs that produce inhibitory functions. Abbreviations: THBS-1, anti-angiogenic protein thrombospondin-1; KRIT1, krev interaction trapped protein 1; PTEN, phosphatase and tensin homolog deleted on chromosome ten; TGF-β, transforming growth factor β; SIRT1, sirtuin 1; FGFR1, fibroblast growth factor receptor 1; Mef2c, myocyte enhancer factor 2C; STAT3, signal transducer and activator of transcription 3; ACLY, proto-oncogene ATP citrate lyase; VEGFR-A, vascular endothelial growth factor receptor A; ZEB1, zinc finger E-box binding homeobox 1; CCR1, chemokine (C-C motif) receptor 1.

For the past few decades, platelet hyperactivation had been elaborated to play important roles in the development and progression of cardiovascular diseases and cancer (105–107). Activated platelets release a variety of vasoactive substances, cytokines and growth factors, promoting platelet-leukocytes crosstalk, mediating the migration of leukocytes, and inducing smooth muscle cell migration and proliferation, further aggravating the damage of vascular-related diseases (108). Platelet secretion also plays multiple roles in cancer fate, including promoting proliferation, resisting cell death, inducing angiogenesis, accelerating invasion and metastasis, and evading immunodetection (109). Activated platelets release and deliver abundant miRNAs as above mentioned; however, few studies have systematic revealed their association and value with clinical diseases. Here, we will explore the possible association of platelet-derived miRNAs with cardiovascular disease and cancer (Tables 3, 4 and Figures 4, 5).

**TABLE 3:** The potential roles of platelet miRNAs on cardiovascular diseases and diabetes.

Diseases	MicroRNA	Target	Associated phenotypes	References
Atherosclerosis	miR-21	MAP3K10	Inhibit macrophage inflammation and atherosclerosis progression	(119)
	miR-223	ICAM-1, NFAT5	Inhibited endothelial inflammation and intra-arterial thrombosis in atherosclerosis; inhibited proliferation and migration of SMCs and atherosclerosis progress	(94) (122)
	miR-34a	Sirt1	Aggravated atherosclerotic plaque development	(121)
	miR-25-3p	Adam 10	Inhibited ox-LDL-induced vascular endothelial inflammation, lipid deposition, and atherosclerosis	(123)
	miR-22-3p	HMGB1	Suppressed the proliferation and migration of SMCs, and neointimal hyperplasia during atherosclerosis	(236)
	Let-7g	PDGF/MEKK1	Decreased atherosclerotic plaques	(237)
	Let-7	IL-6/TGFβR1	Reduced diabetes-associated carotid atherosclerosis	(155)
Myocardial infarction	miR-22-3p	PTAFR	Suppressed the fibrogenesis and collagen deposition of post-MI	(238)
	miR-4306	VEGFA	Reduced macrophage infiltration in cardiac tissue in myocardial infarction mice	(100)
	miR-320b	ICAM-1	Alleviated endothelial inflammation in myocardial infarction mice	(23)
	miR-223-3p	KLF15, PARP-1 RASA1	Protected cardiomyocyte from hypoxia-induced apoptosis and oxidative stress; promoted the proliferation, migration, and differentiation, and aggravated myocardial fibrosis	(239) (128) (127)
	miR-126	HIF-1α VEGFA	Promote angiogenesis in AMI patients	(129)
Hypertension	miR-142-3p	BCLAF1	Promoted endothelial cell proliferation in hypertension	(97)
	miRNA-126	PI3KR2	Prevented microvascular abnormalities in hypertension	(134)
	miR-140-5p	Nrf2/Sirt2	Worsen hypertension and oxidative stress	(240)
	miR-21-3p	HDAC8 ADRA2B	Lowered blood pressure and weakened reduced organ damages in hypertension	(241) (242)
Diabetes	miR-223	P2y12R SMOC1 NLRP3 FOXO1/SOX6 GLUT4	Restrained platelet hyperreactivity associated with diabetes; inhibited endothelial injury induced by high-glucose and high-fat; promoted β-cell proliferation and improved β-cell function in diabetes; Increased glucose metabolism of cardiomyocytes and improved diabetic cardiomyopathy	(158) (230) (154) (243) (244)
	miR-126	ADAM9	Rescued diabetes-induced impairment in efferocytosis of apoptotic cardiomyocytes	(245)
	miR-140	FOXK2	Improved angiogenic dysfunction in diabetes mellitus	(246)
	miR-143	ORP8	Inhibited insulin-stimulated AKT activation and impaired glucose metabolism	(247)
	miR-103b	SFRP4	Early diagnosed DM2	(148)

*ICAM-1, intercellular adhesion molecule 1; NFAT5, nuclear factor of activated T cells 5; Adam 10, a disintegrin and metalloprotease 10; HMGB1, high mobility group box 1; PDGF, platelet derived growth factor; MEKK1, mitogen-activated protein kinase kinase kinase 1; TGFβR1, transforming growth factor beta receptor 1; PTAFR, platelet-activating factor receptor; VEGFA, vascular endothelial growth factor A; KLF15, kruppel like factor 15; PARP-1, poly (ADP-ribose) polymerase 1; RASA1, RAS p21 protein activator 1; BCLAF1, BCL2 associated transcription factor 1; PI3KR2, phosphoinositide-3-kinase regulatory subunit 2; Nrf2, nuclear factor erythroid 2-related factor 2; Sirt2, sirtuin 2; HDAC8, histone deacetylase 8; ADRA2B, adrenoceptor alpha 2b; SMOC1, secreted modular calcium-binding protein 1; NLRP3, nod-like receptor protein 3; Foxo1, forkhead box O1; SOX6, SRY-box transcription factor 6; GLUT4, glucose transporter 4; FOXK2, forkhead box K2; ORP8, oxysterol-binding protein-related protein 8; SFRP4, secreted frizzled related protein 4.*

**TABLE 4:** The potential roles of platelet miRNAs on cancer.

MicroRNA	Target	Associated phenotypes	References
miR-223	Mef2c RhoB EPB41L3	Promoted lung cancer cell invasion	(168) (169) (103)
miR-126	VEGF-A ZEB1 ADAM9 CCR1	Inhibit the progression of ovarian cancer, cervical cancer, prostate cancer, and NSCLC	(172) (173) (174) (175)
miR-24	mt-Nd2	Inhibited growth of both lung and colon carcinoma ectopic tumors	(104)
miR-939	E-cadherin	Induced epithelial-mesenchymal transition and promoted progression of epithelial ovarian cancer	(178)
miR-22	ACLY SIRT1/FGFR1	Inhibited the growth and metastasis in breast cancer, inhibits the angiogenic activities of endothelial cells and consequently NSCLC growth	(180) (187)
Let-7d	HIF1α	Inhibited breast cancer metastasis to the brain	(248)
miR-27b	THBS-1	Enhanced the pro-angiogenic activities	(168)
miR-21	KRIT1 PTEN	Leading to tumor progression	(182) (183)
miR-142	TGF-β	Leading to decreased growth and metastasis of hepatocellular carcinoma by antagonizing angiogenesis	(186)

*HIF1α, hypoxia inducible factor 1 subunit alpha; VEGF-A, vascular endothelial growth factor A.*

#### Platelet-Derived MicroRNAs and Atherosclerosis

Atherosclerosis (AS) is a chronic cardiovascular disease that underlies the pathology of cerebral infarction and coronary heart disease (110). The occurrence and development of AS involve in a series of pathological and physiological processes, mainly including vascular endothelial damage, inflammatory cell and lipid infiltration, platelet activation, and intimal thickening (111–113). Platelets are considered to be important contributors to atherosclerosis for their ability to induce inflammatory cascades. Activated platelets lead to vascular damage through the expression and release of inflammatory mediators and promote the activation and degeneration of endothelial cells to form atherosclerosis and vascular thrombotic lesions (114). They also promote intercellular communication and adhesion between blood cells and the vessel wall, proliferation of SMCs and chemotaxis of foam cells (115, 116).

As mentioned above, platelet-derived miRNAs not only regulate platelet function, but also participate in endothelial cell function and SMC proliferation, suggesting that they may play a significant role in the occurrence and development of atherosclerosis. Several studies have found platelet-derived miRNAs changed in the circulation of atherosclerotic patients. In study performed by Sondermeijer group, circulating miR-624 and miR-340 are found to be significantly elevated in patients with CAD as compared to healthy controls (117). However, the expression of miR-126 and miR-223 in platelets were reduced in AS patients. Among them, miR-126 expression level was proven to have a negative correlation with plaque morphology and coronary stenosis (118). The mechanism may involve the targeted regulation of MAP3K10 by miR-21 to inhibit macrophage inflammation and atherosclerosis progression (119). In a atherosclerosis animal model, more miRNAs such as miR-19a, −21, −126, −26b, −92a, −155, −204, −210, −221, −222, and −34a are reported to be delivered by PMVs and contribute to the richness of circulating miRNAs (120). Elevated miR-34a aggravated atherosclerotic plaque development by inhibiting Sirt1 signaling in an atherosclerosis mouse model (121). Other demonstrated the roles of platelet-derived miRNAs in many key links of atherosclerosis development. MiR-223 released by thrombin-activated PMVs inhibits ICAM-1 expression by regulating NF-κB and MAPK pathways and is protective against atherosclerosis and endothelial inflammation (94). MiR-223 also inhibits the platelet-derived growth factor-BB (PDGF-BB)-induced proliferation and motility of human aortic smooth muscle cells by targeting NFAT5 and exhibits potential therapeutic effects for atherosclerosis (122). Platelet-derived exosomes overexpressing miR-25-3p attenuate coronary vascular endothelial cell inflammation induced by oxidized low density lipoprotein *via* targeting NF-κB/Adam10 Pathway in ApoE-/- mouse models of atherosclerosis (123). However, whether other differentially expressed platelet miRNAs are also involved in the occurrence and development of atherosclerosis remains unclear.

#### Platelet-Derived MicroRNAs and Myocardial Infarction

The rupture of unstable plaque leading to rapid activation and aggregation of circulating platelets, and following thrombosis, is the main mechanism of myocardial infarction. Several selected platelet miRNAs were found to significantly change in acute myocardial infarction (AMI) and become indicators of thrombosis. Candidate platelet miR-21 and miR-126 were significantly descended in patients with AMI compared with the controls while the results of platelet miR-150 and miR-223 were opposite. Hromadka et al. found that miR-126 and miR-223 were potential independent predictors of thrombotic events and recommended for ischemic risk stratification after AMI (124). The expression levels of platelet miR-587 were relatively higher in AMI patients than unstable angina (UA) and control groups and showed positive association with the degree of coronary stenosis (125). These results suggest that platelet-derived miRNAs may induce atherosclerotic plaque instability or thrombosis besides platelet function. Elgheznawy et al. found that in a mouse FeCl3-induced arterial thrombosis model, miR-223 deficiency increased thrombus size after FeCl3 carotid treatment, and increased embolization after laser-induced vascular injury of the small dorsal skin (67). MiR-223 directly targets kindlin3, an integrin-binding protein, and FXIII-A, both of which contribute to thrombus formation. Therefore, platelets unexpectedly antagonize the formation of thrombi by releasing specific miRNAs, such as miR-223, when they are abnormally activated in the early stage of MI, through above mechanisms. Additionally, miR-223 has also been found to exhibit contradictory direct cardiac effects in myocardial infarction, including protecting cardiomyocytes from ischemic injury, promoting fibroblast proliferation and collagen formation (126–128). Of course, platelet miRNAs also influence platelet activity by targeting its surface receptors, such as miR-223/P2Y12 and miR-126/ADAM9, accounting for their potential behaviors during thrombosis (27, 68). Besides, inflammatory response accelerating myocardial fibrosis after myocardial infarction is closely related to the deterioration of cardiac function and long-term prognosis. MiR4306 from PMVs noticeably inhibited macrophage infiltration in cardiac tissue in myocardial infarction mice, which may also restrain the progression of post-infarction remodeling (100). Endothelial cells expressing ICAM-1 triggering the adhesion and migration of inflammatory cells to the damaged myocardium, has been found to be regulated by platelets releasing miR-320b (23) and miR-223 (122). Additionally, exosomes containing miR-126 extracted from AMI patients promote angiogenesis by increasing HIF-1α and VEGFA expression (129).

#### Platelet-Derived MicroRNAs and Hypertension

Hypertension is a complex, multifactorial disease, and its occurrence and development have definite relationships with miRNA regulatory network (130). Marketou et al. assessed platelet-derived miRNAs in 82 patients with essential hypertension and 28 healthy individuals and found that miR-22 and −223 levels are significantly decreased in hypertension and negatively correlated with SBP levels (131). Additionally, let-7 is reported to have a positive correlation with carotid intima-media thickness in patients with essential hypertension (132) while miR-21 has a negative association with arterial stiffness (133). These results suggest that platelets are involved in the formation and progress of hypertension by delivering miRNAs, although the mechanisms seem not very clear. Abnormal proliferation of endothelial cells (EC) induced by pathologic factors contributes to vascular remodeling in hypertensive conditions. MiR-142-3p from PMVs enhanced EC proliferation by downregulating the expression of BCLAF1 (97). In a spontaneously hypertensive rat (SHR) model, miRNA-21 levels were increased by 36% levels while miRNA-126 levels were reduced by 29%. Both of them are abundant in platelets. Aerobic exercise training reversed their changes and prevented microvascular abnormalities in hypertension *via* increasing the expression of anti-apoptotic protein Bcl-2 and inhibiting the expression of anti-angiogenic regulator PI3KR2 (134). Thus, regulating endothelial cell proliferation in hypertensive vascular remodeling is one of the mechanisms to explain the potential role of platelet-derived miRNAs. Additionally, PMVs from thrombin-stimulated platelets transferring miR-223, −339, and −21 into SMCs were shown to inhibit their proliferation by downregulating the expression of PDGFRb (98). However, whether their proliferation effects on SMCs is enough to change the phenotype of hypertension deserves further study.

#### Platelet-Derived MicroRNAs and Diabetes

Diabetes mellitus is a serious threat to human lifespan worldwide, resulting in a 2–4 times increase in mortality compared with non-diabetic subjects (135, 136). Cardiovascular complications are the leading cause of death from diabetes, accounting for 50–75% of deaths (137, 138). It has been widely accepted that diabetes are prone to pro-thrombic condition by increasing coagulation activity, impaired fibrinolysis, endothelial dysfunction, and platelet hyper-reactivity. Although the detail mechanism of hypercoagulable state in diabetes remain unclear, hyperactivation, and endothelial dysfunction observed in diabetes contribute to this pathological process (139). In 1993, Nomura et al. reported that platelets were activated in diabetic patients and the microparticles released from them also increased in plasma (140, 141). Activated platelets are able to modulate the function of ECs and SMCs, contributing to both the initiation and progression of atherosclerosis, and even the ensuing atherothrombotic sequelae (142, 143). Recently, it has been reported that platelets have been found to be a major source of circulating miRNAs and specific miRNAs from platelets changed in diabetes, suggesting that platelets-derived miRNAs may be potential predictive markers and therapeutic targets.

Fejes et al. reported that hyperglycemia suppresses miRNAs expression in platelets. They found the expression of miR-223, miR-26b, miR-140, and miR-126 in mature platelets had been significantly inhibited in T2DM subjects (144), which at least partly because hyperglycemia decreased platelet Dicer activity (145). Additionally, miR-26b and miR-140 showed direct target on the gene SELP and miR-223 and miR-126 inhibited the expression of P2Y12 in platelets (144, 146). Similarly, Elgheznawy et al. found human subjects and mice with diabetes showed decreased levels of platelet miR-142, miR-143, miR-155, and miR-223, which possibly related to the inhibition of Dicer enzyme. Using calpain inhibitors to prevent the loss of platelet Dicer in diabetic mice can save the decrease of platelet-miRNAs and increase their target genes (67). Besides miR-223, the expression of miR-146a was also downregulated by hyperglycemia intervention, resulting in subsequent platelet activation in patients with diabetes. Moreover, low level of miR-223 and miR-146a in patients plasma with diabetes increases the risk of ischemic stroke (147). Platelet-derived miR-103b is significantly downregulated and also suggested as a novel biomarker for the early diagnosis of T2DM (148). Additionally, decreased miR-30c level induced by hyperglycemia promotes thrombus formation in T2DM by increasing the expression of plasminogen activator inhibitor-1 (PAI-1) (149).

The change of platelet-derived miRNAs contributed to diabetes-associated vascular lesion. Endothelial dysfunction in patients with T2DM is well recognized, resulting in the vascular system susceptible to thrombotic and atherosclerotic effects (150). Recent plasma miRNA analysis confirmed that the downregulation of miR-126 in a group of diabetic patients are partially responsible for vascular damage in diabetic patients (151, 152). Elevated plasma miR-21 in diabetic patients was found to decrease the production of reactive oxygen species and inflammatory cytokines in vascular endothelial cells and reduce the area of atherosclerotic plaque by targeting the degradation of ADAM10 mRNA (153). Additionally, miR-223-3p improves the injury of cardiac microvascular endothelial cells from diabetic mice by targeting the expression of NLRP3 (154). Let-7, an abundant miRNA in PMVs, is decreased in human and mice carotid plaques with diabetes, and promotes the inflammatory phenotypes of SMCs including proliferation, migration, monocyte adhesion, and NF-κB activation (155).

Targeting platelet-derived miRNAs appears to confer some protective effects in diabetes. For example, aerobic training improves platelet function in type 2 diabetic patients *via* increasing miRNA-130a and decreasing the target gene GPIIb (156). Long-term moderate-intensity aerobic training increased miRNA-223 expression, leading to decreased expression of P2Y12 receptor and platelet activity, which may be one of internal mechanisms for reducing the occurrence of atherothrombotic events in T2DM patients (157, 158). Additionally, inhibiting platelet activation by aspirin reduced levels of circulating miR-126 (159), which may protect endothelial from inflammation.

#### Platelet-Derived MicroRNAs and Cancer

Cancer is the leading cause of death following CVDs and shows close associations with CVDs. Tumors are often accompanied by overactive platelets and hypercoagulable state, as well as endothelial proliferation and angiogenesis in the tumor microenvironment, overlapping some pathogenesis with many cardiovascular diseases (160, 161). Long-term platelet inhibition in cardiovascular disease is considered to regulate tumor fate by alleviating chronic inflammation and endothelial angiogenesis (162, 163). Additionally, tumor therapy often leads to cardiovascular disease, which is well-known as cardio-oncology (164). Therefore, platelet function and its derived miRNAs may be the common markers for diagnosis and common targets for treatment about these two diseases.

In fact, platelets are active participants in all steps of tumorigenesis, including tumor growth, angiogenesis, and metastasis. On the one hand, PMVs transfer platelet miRNAs to vascular endothelial cells and are associated with enhanced tumor metastasis and cancer progression. On the other hand, due to the high permeability of the vascular system of solid tumors, PMVs can penetrate the blood vessels and enter the tumor microenvironment to directly transfer platelet miRNAs into tumor cells, thus regulating gene expression in tumor cells and tumor progression (165, 166). Subcutaneously implanted tumors with platelet miRNA knockout in mice aggravated sarcomatoid growth and progress, verified the tumor-promoting effects of platelet miRNAs (167).

In different cancer cells, PMVs derived miRNAs target different oncogenes and tumor suppressor genes. It has been reported that miR-223 enhances breast cancer invasion by inhibiting the expression of myocyte enhancer factor 2C (Mef2c) and increases the progression of gastric cancer by specifically targeting RhoB (168, 169). It has also been reported that miR-223 directly targets the 3′-UTR of the tumor suppressor EPB41L3, which is the most upregulated gene in recurrent tumors (170). Increased miR-223 was observed in platelets and PMVs from NSCLC patients and effectively delivered to human lung cancer cell A549 *via* PMVs (103), thus facilitating the invasion of A549. Physiological delivery of platelets miR-223 and miR-126 altered the phenotypes of breast cancer cells, including cell cycle arrest, migration inhibition, and increased responsiveness to cisplatin (171). MiR-126 can inhibit the progression of some cancers (including ovarian cancer, cervical cancer, prostate cancer, and NSCLC) *via* negative control of numerous validated targets such as VEGF-A, ZEB1, ADAM9, and CCR1 (172–175). In some instances, however, miR-126 supports cancer progression *via* inhibiting STAT3-mediated tumor apoptosis (176, 177). MiR-24 in PMVs inhibits the growth of ectopic tumors of lung and colon cancer by targeting mitochondrial protein mt-Nd2. Blocking miR-24 in tumor cells accelerates their growth *in vivo* and eliminates the inhibitory effect of PMVs on tumor growth (104). In ovarian cancer cells, miR-939 delivered by PMVs induces mesenchymal transformation of epithelial cells and cancer progression by inhibiting the expression of e-calponin expression (178). In small-cell lung cancer (SCLC), overexpression of miR-24-3p blocked the autophagy process by targeting autophagy-associated gene 4A (ATG4A), finally enhancing the sensitiveness of SCLC cells to combined chemotherapy (etoposide and cisplatin) (179). In breast cancer, miR-22 inhibited the growth and metastasis by downregulating the expression of the proto-oncogene ATP citrate lyase (ACLY) (180). In solid tumors, PMVs interact with tumor cells by directly transferring platelet-derived miRNAs and inhibit the growth of ectopic tumors in colon and lung cancers by downregulating TC genes and inducing tumor cell apoptosis (104).

Platelet miRNAs also transfer to vascular endothelial cells and regulate tumor development and drug resistance by enhancing or inhibiting angiogenesis. When PMVs were co-cultured with HUVECs on extracellular matrix gels, PMVs-derived let-7a was adopted to endothelial cells and induced robust capillary like structure formation by promoting the release of pro-angiogenic regulators and reducing the expression of anti-angiogenic protein thrombospondin-1 (THBS-1) (96). In another *in vitro* experiment, THBS-1 expression was inhibited by transfection with elevated platelet miR-27b, which was subsequently enhanced the pro-angiogenic activities of platelet (181). Exosomal miR-21 in the tumor microenvironment had been widely known as a strong proangiogenic factor *via* targeting krev interaction trapped protein 1 (KRIT1) and PTEN (182, 183), finally leading to tumor progression. However, miR-223 is identified as an antiangiogenic miRNA by targeting β1 integrin (184), thereby promoting resistance to cetuximab in head and neck squamous cell carcinoma (185). Moreover, miR-142 was found to directly target and inhibit transforming growth factor β (TGF-β), leading to decreased growth and metastasis of hepatocellular carcinoma by antagonizing angiogenesis (186). MiR-22 also acts as a potent angiogenesis inhibitor that inhibits the angiogenic activities of endothelial cells and consequently NSCLC growth through targeting SIRT1 and FGFR1 (187).

### Platelet Inhibitors and Platelet-Derived MicroRNAs

Antiplatelet drugs are considered as the cornerstone for the prevention and treatment of atherothrombotic diseases, and have saved numerous lives since inception. However, a considerable number of patients receiving standard antiplatelet medication still exhibit high levels of platelet activation, increasing their risk of progression and recurrence of cardiovascular events (188, 189). Several possible mechanisms were proposed to explain the phenomenon, such as genetic polymorphisms, drug–drug interactions, or high on-treatment platelet reactivity (HTPR) (190, 191). However, identifying patients who has inadequate response to state-of-the-art antiplatelet treatment remains a challenge. Thus, real-time monitoring of platelet activity seems to be more accessible and important for patients with atherothrombotic diseases and can ideally guide personalized antiplatelet treatment. Current platelet function tests are measured *ex vivo* and susceptible to interference by many confounding factors, bringing obvious limitations for guiding treatment decisions. As mentioned above, platelet activation leads to the production of PMVs carrying abundant miRNAs, which in turn change platelet function and affect protein expression in other cells upon internalization. Antiplatelet drugs including COX inhibitors and P2Y12 receptor inhibitors were found to significantly change the expression of platelet-derived miRNAs (36, 192, 193). Circulating platelet miRNAs are relatively stable and convenient for detection *in vivo*, making them potential and reliable markers for monitoring platelet activity and antiplatelet response.

Willeit et al. firstly examined the responsiveness of platelet-derived miRNAs to platelet inhibition. They introduced that plasma levels of platelet miRNAs, including miR-126, miR-150, miR-191, and miR-223, are significantly reduced by aspirin and prasugrel treatment (91). Another recent study found that the levels of plasma miR-223 and miR-197 from platelets are significantly downregulated in subjects treated with clopidogrel or ticagrelor when compared with health controls (194). Acute coronary syndrome patients treated with clopidogrel alone resulted in 2-fold reduction in miR-223, 1.8-fold reduction in miR-130, and 4.1-fold reduction in miR-126 (195). Apparently, inconsistent miRNA alterations in different researches may be attributed to the kind of antiplatelet reagents, which results in varied degree of platelet inhibition. A recent study reported that plasma platelet miRNAs (such as miR-126, miR-150, and miR-223) were significantly reduced in ACS patients who completed the replacing treatment from clopidogrel to another more potent antiplatelet agent ticagrelor, in proportion to the degree of platelet inhibition (196). Additionally, monotherapy with potent P2Y12R inhibition prasugrel in T2DM reduced the levels of miR-24, miR-191, miR-197, and miR-223 when compared with aspirin treatment (197). Therefore, consensus on which miRNA is the best biomarker for the response to antiplatelet therapy has not yet been reached. Circulating miR-223 and miR-126 are expected to be the options since they reached similar conclusion in multiple studies (198–201).

Plasma level of platelet-derived miRNAs can also be used as a marker of antiplatelet insensitivity or resistance, also named high on-treatment platelet reactivity (HTPR). Kok et al. proposed that miR-19b-1-5p is a suitable marker of platelets insensitive to aspirin (202). MiR-365-3p is found to be positively correlated with HTPR in coronary artery disease patients (203). Additionally, platelets in ACS patients with HTPR exhibit upregulation of miR-204-5p after dual antiplatelet therapy (204). Lower expression of miR-126, miR-130, and miR-223 is also been observed in the ACS patients with high platelet reactivity (HPR) to clopidogrel than those with low platelet reactivity (LPR) (195). A more recent study also reported that increased expression of miR-24-3p, miR-142-3p, and miR-411-3p was positively correlated with clopidogrel resistance (CR) in CAD patients (205). Similarly, miR-29, miR-34, miR-126, miR-142, and miR-223 are also reported to be novel biomarkers for P2Y12 inhibitor resistance prediction (206). Although the mechanisms of antiplatelet resistance are complex, the relationship between plasma miRNAs and platelet resistance may be explained by their regulation on platelet surface proteins. Liu et al. found that miR-34b-3p overexpression inhibited the expression of thromboxane A synthase 1 (TBXAS1), leading to the enhanced antiplatelet efficiency of aspirin (207). The downregulated miR-107 and miR-223 in the HPR group are negatively correlated with P2Y12 expression, indicating that platelet miR-107 and miR-223 possibly mediated CR by inhibiting P2Y12 expression (208, 209). A more recent study also found that platelet miR-15b promoted platelet insensitivity in patients undergoing PCI because it suppressed Bcl-2 protein expression and enhanced platelet apoptosis (210).

In general, on the one hand, platelet-derived miRNAs are significantly altered by antiplatelet drugs and become potential indicators of platelet activity level. On the other hand, changes in platelet miRNA levels may in turn affect their response to antiplatelet drugs by altering the expression of platelet-activated receptors.

### Clinical Significances of Platelet-Derived MicroRNAs in Cardiovascular and Neoplastic Diseases

As mentioned above, platelets are excessively activated in various vascular diseases, diabetes and tumors, and secrete many cell-specific miRNAs through PMVs. These miRNAs remain stable in peripheral blood, allowing a convenient detection, bringing them potential perspectives of early clinical diagnosis for platelet-related diseases (Table 5). Additionally, platelet miRNAs control and regulate the biological functions of themselves and other neighboring cells, participating in the occurrence and development of cardiovascular diseases and tumors, becoming novel potential targets for treatment.

**TABLE 5:** Changes of platelet miRNAs in cardiovascular disease and cancer.

Changed microRNAs	Disease	References
miR-340↑, miR615-5p↑, miR-545:9.1↑, miR-451↑, miR-454↑, miR-624↑, miR-624↑, miR-12801↓	Premature coronary artery disease	(117)
miR-150↑, miR-223↑, miR-21↓, miR-126↓	STEMI patients	(215)
miR-142-3p↑, miR-107↑, miR-338-3p↑, miR-223-3p↑, miR-21-5p↑, miR-130b-3p↑, miR-301a-3p↑, miR-221-3p↑	ACS patients	(219)
miR186-5p↓, miR185-5p↓, miR20a-5p↓, miR942↓, miR127-3p↑, miR221-3p↑, miR483-5p↑, miR146a-5p↑	Acute coronary syndrome	(249)
miR-150↑	Heart failure with atrial fibrillation	(250)
miR-144↑, miR-146a↓, miR-223↓	Diabetes mellitus type 2 patients with ischemic stroke	(221) (147)
miR-223↑	NSCLC patients	(103)
miR-34c-3p↑, miR-18a-5p↑	Nasopharyngeal carcinoma patients	(226)

#### Platelet-Derived MicroRNAs as Potential Markers of Cardiovascular Diseases

Considering the high mortality and morbidity of cardiovascular diseases and the lack of timely diagnosis, the discovery of novel predictive biomarkers is necessary. However, current diagnostic techniques based on electrocardiogram and troponin, are severely limited because they may be non-specifically altered in certain diseases, such as myocarditis and secondary myocardial damage. Platelet hyperactivation is another important clinical feature during acute thrombotic events (211), yet no corresponding test accurately reflects its states. Platelet-derived miRNAs have been reported to be biomarkers for platelet activation and are expected to be diagnostic and/or prognostic biomarkers for cardiovascular disease.

MiR-1, one of platelet-rich miRNAs, was found to increase rapidly and peak within 2 h after the onset of cardiac infraction and positively correlated with serum creatine kinase MB (CK-MB) levels (212). Another study showed that both increased miR-1 and miR-29b were associated with the decreased parameters of cardiac function (such as LVEDV and LVEF) in patients suffered AMI, indicating their potential predictive roles for adverse ventricular remodeling (213). MiR-126 and miR-223 were the most frequently investigated platelet miRNAs and it is well established that they are significantly reduced in CVD patients, making them an indication for the presence of cardiovascular diseases (131, 214). Five candidate platelet miRNAs, including miR-1, miR-21, miR-126, miR-199, and miR-233, were compared in patients with ST segment elevation myocardial infarction (STEMI) and healthy volunteers. Among them, only miR-126 exhibited correlation with plasma cTnI and was expected to be a potential novel biomarker for STEMI (215). Moreover, miR-126 also is proved as a strong and independent predictor of long-term all-cause mortality among patients with T2DM (216) and patients with venous thromboembolism (217). In a large patient cohort with CAD, Serum miR-223 as well as miR-197 levels were found to be predictors for cardiovascular death (218). Additionally, the predictive accuracy for one-year comprehensive ischemic endpoint was significantly increased when miR-223 and miR-126/miR-223 ratios were served as predictors and added into the model calculating the ischemic risk (124). However, another study assessed potential biomarkers of ACS based on the miRNA profiles of platelets and found that eight platelet miRNAs were markedly elevated in ACS patients and associated with platelet reactivity and functionality. Among them, miR-142-3p is the only potential biomarker confirmed to accurately predict the risk of ACS (219).

Circulating platelet miRNAs may also be sensitive and specific biomarkers for ischemic stroke. In T2DM patients with ischemic stroke, the miR-144 level in platelets increased significantly (220). However, the expression of platelet miR-223 was significantly reduced in these subjects when compared with T2DM patients in without thromboembolic complications (221). Similarly, Duan et al. found that the expressions of platelet miR-223 and miR-146a was obviously lower in diabetic and ischemic stroke patients than in healthy donors (147). Additionally, the expression level of these two miRNAs was correlated with blood platelet activation rates.

#### Platelet-Derived MicroRNAs as Potential Markers of Cancer

Currently, the diagnosis of cancer mainly depends on clinical manifestations, radiological and biochemical tests and pathological analysis. Although biopsies are the current gold standard for cancer diagnosis, the information obtained from individual biopsies provides a limited snapshot of tumors in space and time, and is not suitable for repeated sampling. Therefore, the liquid biopsy is considered a promising tool for early detection and subsequent monitoring of cancer (222, 223). Platelets are an important component of blood, and their ability to store and release numerous miRNAs to the environment, which enables them to reflect different disease states, early diagnose tumors, predict prognosis, monitor response to treatment, and detect disease recurrence and metastasis (224).

Platelet miRNAs were significantly altered in tumor patients compared with healthy donors (225). For example, platelet derived miR-223 was specifically overexpressed in NSCLC patients than healthy subjects (103). Additionally, tumor-educated platelet was reported to express higher levels of receiving miR-34c-3p and miR-18a-5p from nasopharyngeal carcinoma (NPC) cells, making them potential makers for NPC diagnosis (226). The positive rates for NPC diagnosis based on platelet miR-34c-3p and miR-18a-5p were 93.8 and 87.5%, respectively, significantly higher than these based on Epstein-Barr virus DNA (66.7%) (226). Moreover, a large prospective trial by Best et al. in 2015 showed that the RNA profile of tumor-affected platelets was different from that of healthy individuals, and identified the location of the primary tumor with 96% accuracy and 71% accuracy (227). Therefore, platelets and their derived miRNAs may be a potential source for the development of oncology blood biomarkers.

Platelet miRNAs can also be used to evaluate efficacy and patient outcomes. miR-21, miR-25, miR-19b, and miR-146a and in patients with NSCLC may be potential indicators to predict response to platinum-based treatments (228). Gasperi et al. reported that ω6/ω3-PUFA supplementation enhanced platelet antitumor activities by promoting PMVs derived miRNA (miR-223 and miR-126) delivery into breast cancer. These two miRNAs inhibited the cancer proliferation and metastasis and increased the sensitivity to cisplatin once internalized by breast cancer cells in a dose-dependent manner (171). Additionally, cardiovascular damage caused by tumor treatment contributes to the increasing mortality of cancer (229). Platelet-derived miRNAs may also become potential markers in the field of cardio-oncology. The levels of miR-223-3p decreased in radiation-induced heart in a time-dependent manner and exhibits potential protection against radiation-induced cardiac toxicity (126).

However, we still face many difficulties in trying to apply platelet miRNAs to the clinic. The primary problem is how to solve the potential infection of plasma miRNA background on platelet miRNA. To avoid this problem, limiting miRNA measurements to PMVs has been recommended, but this would imply a greater workload and a more complex workflow. Future studies will need to explore convenient, economical and accurate detection methods.

#### Treatment Prospect

Alterations in tissue-specific or cell-specific miRNA expression and their regulation of pathogenic genes under different disease conditions provide a theoretical basis for the use of miRNA technologies to treat diseases. In the past few years, chemically modified oligonucleotides called antagomirs have been developed to silence specific endogenous miRNAs *in vivo* and *in vitro*. Intravenous systemic administration of antagomirs has been widely demonstrated to effectively and specifically recognize and inhibit the activity of target miRNAs in a sequence complementary binding manner in many cells. Several miRNA-targeted therapies have entered the stage of clinical development. For example, a mimetic tumor suppressor miR-34 has reached phase I clinical trials for the treatment of cancer. Additionally, a biological bead targeting miR-122 has entered the phase II trials for the treatment of hepatitis. However, drug delivery issues remain a barrier to their therapeutic use, particularly for targeting miRNAs in cardiovascular disease states. The properties of platelet endocytosis and transport of circulating RNAs have given us new solutions. Vesicle storage coupled with the relatively long half-life of miRNAs could allow miRNAs to persist in circulation for a long time, thus effectively silencing targets in various organ systems.

## Conclusion

Overall, platelets are involved in several normal physiological processes such as hemostasis, inflammation, vascular repair, and generation, and play a role in diseases such as atherosclerosis, diabetes and cancer. Several studies have determined that activated platelets release miRNA-rich PMVs to regulate other cellular functions. With the intensive study of platelet-derived miRNAs, we realize that platelet miRNAs have the potential to be excellent diagnostic tools covering multiple pathological mechanisms simultaneously. Studies have shown that platelet miRNAs can directly or indirectly reflect platelet activity, thus indicating the emergence of pathological states at an early stage or assessing efficacy after treatment. We can also exploit the organ specificity of some miRNAs within platelets to enhance the effect of existing drugs or to find new therapeutic targets. In conclusion, studying platelet-derived miRNAs can be of great benefit to patients by helping to modify the use of existing drugs and finding new drug targets, as well as for assessing treatment efficacy and patient prognosis. Although there are still questions to be answered, platelet-associated miRNAs are promising biomarker candidates.

## Author Contributions

QL and LW wrote the manuscript, prepared the tables, and drew the figures. JD helped to process the figures. LW and YW designed the experiments, guided this study, and revised the manuscript. MD, LL, QF, and DW checked this manuscript. All authors contributed to this article and approved the submitted manuscript.

## Conflict of Interest

The authors declare that the research was conducted in the absence of any commercial or financial relationships that could be construed as a potential conflict of interest.

## Publisher’s Note

All claims expressed in this article are solely those of the authors and do not necessarily represent those of their affiliated organizations, or those of the publisher, the editors and the reviewers. Any product that may be evaluated in this article, or claim that may be made by its manufacturer, is not guaranteed or endorsed by the publisher.

## References

[1] Roth G Mensah G Johnson C Addolorato G Ammirati E Baddour L Global burden of cardiovascular diseases and risk factors, 1990-2019: update from the GBD 2019 study. *J Am Coll Cardiol.* (2020) 76:2982–3021. 10.1016/j.jacc.2020.11.010 33309175 PMC7755038

[2] Masaebi F Salehi M Kazemi M Vahabi N Azizmohammad Looha M Zayeri F. Trend analysis of disability adjusted life years due to cardiovascular diseases: results from the global burden of disease study 2019. *BMC Public Health.* (2021) 21:1268. 10.1186/s12889-021-11348-w 34187450 PMC8244206

[3] Roth G Mensah G Fuster V. The global burden of cardiovascular diseases and risks: a compass for global action. *J Am Coll Cardiol.* (2020) 76:2980–1. 10.1016/j.jacc.2020.11.021 33309174

[4] Fitzmaurice C Abate D Abbasi N Abbastabar H Abd-Allah F Abdel-Rahman O Global, regional, and national cancer incidence, mortality, years of life lost, years lived with disability, and disability-adjusted life-years for 29 cancer groups, 1990 to 2017: a systematic analysis for the global burden of disease study. *JAMA Oncol.* (2019) 5:1749–68. 10.1001/jamaoncol.2019.2996 31560378 PMC6777271

[5] Jemal A Bray F Center M Ferlay J Ward E Forman D. Global cancer statistics. *CA Cancer J Clin.* (2011) 61:69–90. 21296855 10.3322/caac.20107

[6] Murphy S Ibrahim N Januzzi J. Heart failure with reduced ejection fraction: a review. *JAMA.* (2020) 324:488–504. 32749493 10.1001/jama.2020.10262

[7] Hirsch F Scagliotti G Mulshine J Kwon R Curran W Wu Y Lung cancer: current therapies and new targeted treatments. *Lancet.* (2017) 389:299–311. 27574741 10.1016/S0140-6736(16)30958-8

[8] Rowley J Schwertz H Weyrich A. Platelet mRNA: the meaning behind the message. *Curr Opin Hematol.* (2012) 19:385–91. 10.1097/MOH.0b013e328357010e 22814651 PMC3670814

[9] Stojkovic S Nossent A Haller P Jäger B Vargas K Wojta J MicroRNAs as regulators and biomarkers of platelet function and activity in coronary artery disease. *Thromb Haemost.* (2019) 119:1563–72. 10.1055/s-0039-1693702 31421643

[10] Estevez B Du X. New concepts and mechanisms of platelet activation signaling. *Physiology (Bethesda).* (2017) 32:162–77. 10.1152/physiol.00020.2016 28228483 PMC5337829

[11] Li Z Delaney M O’Brien K Du X. Signaling during platelet adhesion and activation. *Arterioscler Thromb Vasc Biol.* (2010) 30:2341–9. 21071698 10.1161/ATVBAHA.110.207522 PMC3085271

[12] Wu L Zhao F Dai M Li H Chen C Nie J P2y12 receptor promotes pressure overload-induced cardiac remodeling via platelet-driven inflammation in mice. *Hypertension.* (2017) 70:759–69. 10.1161/HYPERTENSIONAHA.117.09262 28827474

[13] Khodadi E. Platelet function in cardiovascular disease: activation of molecules and activation by molecules. *Cardiovasc Toxicol.* (2020) 20:1–10. 10.1007/s12012-019-09555-4 31784932

[14] Dib P Quirino-Teixeira A Merij L Pinheiro M Rozini S Andrade F Innate immune receptors in platelets and platelet-leukocyte interactions. *J Leukocyte Biol.* (2020) 108:1157–82. 10.1002/JLB.4MR0620-701R 32779243

[15] Gleissner C. Platelet-derived chemokines in atherogenesis: what’s new? *Curr Vasc Pharmacol.* (2012) 10:563–9. 10.2174/157016112801784521 22338571

[16] Smeda M Przyborowski K Stojak M Chlopicki S. The endothelial barrier and cancer metastasis: does the protective facet of platelet function matter? *Biochem Pharmacol.* (2020) 176:113886. 10.1016/j.bcp.2020.113886 32113813

[17] Brill A Dashevsky O Rivo J Gozal Y Varon D. Platelet-derived microparticles induce angiogenesis and stimulate post-ischemic revascularization. *Cardiovasc Res.* (2005) 67:30–8. 10.1016/j.cardiores.2005.04.007 15878159

[18] Coppinger J Cagney G Toomey S Kislinger T Belton O McRedmond J Characterization of the proteins released from activated platelets leads to localization of novel platelet proteins in human atherosclerotic lesions. *Blood.* (2004) 103:2096–104. 10.1182/blood-2003-08-2804 14630798

[19] Ed Nignpense B Chinkwo K Blanchard C Santhakumar A. Polyphenols: modulators of platelet function and platelet microparticle generation? *Int J Mol Sci.* (2019) 21:146. 10.3390/ijms21010146 31878290 PMC6981839

[20] Plé H Landry P Benham A Coarfa C Gunaratne P Provost P. The repertoire and features of human platelet microRNAs. *PLoS One.* (2012) 7:e50746. 10.1371/journal.pone.0050746 23226537 PMC3514217

[21] Dangwal S Thum T. MicroRNAs in platelet biogenesis and function. *Thromb Haemost.* (2012) 108:599–604. 22782083 10.1160/TH12-03-0211

[22] Neu C Gutschner T Haemmerle M. Post-transcriptional expression control in platelet biogenesis and function. *Int J Mol Sci.* (2020) 21:7614. 10.3390/ijms21207614 33076269 PMC7589263

[23] Gidlöf O van der Brug M Ohman J Gilje P Olde B Wahlestedt C Platelets activated during myocardial infarction release functional miRNA, which can be taken up by endothelial cells and regulate ICAM1 expression. *Blood.* (2013) 121:3908–17. 10.1182/blood-2012-10-461798 23493781

[24] Bartel D. MicroRNAs: genomics, biogenesis, mechanism, and function. *Cell.* (2004) 116:281–97. 10.1016/s0092-8674(04)00045-5 14744438

[25] Krol J Loedige I Filipowicz W. The widespread regulation of microRNA biogenesis, function and decay. *Nat Rev Genet.* (2010) 11:597–610. 10.1038/nrg2843 20661255

[26] Kim V. MicroRNA biogenesis: coordinated cropping and dicing. *Nat Rev Mol Cell Biol.* (2005) 6:376–85. 10.1038/nrm1644 15852042

[27] Landry P Plante I Ouellet D Perron M Rousseau G Provost P. Existence of a microRNA pathway in anucleate platelets. *Nat Struct Mol Biol.* (2009) 16:961–6. 10.1038/nsmb.1651 19668211 PMC2911476

[28] Edelstein L Bray P. MicroRNAs in platelet production and activation. *Blood.* (2011) 117:5289–96. 21364189 10.1182/blood-2011-01-292011 PMC3109704

[29] Edelstein L McKenzie S Shaw C Holinstat M Kunapuli S Bray P. MicroRNAs in platelet production and activation. *J Thromb Haemost.* (2013) 11:340–50. 23809137 10.1111/jth.12214

[30] Rowley J Oler A Tolley N Hunter B Low E Nix D Genome-wide RNA-seq analysis of human and mouse platelet transcriptomes. *Blood.* (2011) 118:e101–11. 10.1182/blood-2011-03-339705 21596849 PMC3193274

[31] Cecchetti L Tolley N Michetti N Bury L Weyrich A Gresele P. Megakaryocytes differentially sort mRNAs for matrix metalloproteinases and their inhibitors into platelets: a mechanism for regulating synthetic events. *Blood.* (2011) 118:1903–11. 10.1182/blood-2010-12-324517 21628401 PMC3158719

[32] Clancy L Beaulieu L Tanriverdi K Freedman J. The role of RNA uptake in platelet heterogeneity. *Thromb Haemost.* (2017) 117:948–61. 10.1160/TH16-11-0873 28276570

[33] Risitano A Beaulieu L Vitseva O Freedman J. Platelets and platelet-like particles mediate intercellular RNA transfer. *Blood.* (2012) 119:6288–95. 10.1182/blood-2011-12-396440 22596260 PMC3383198

[34] Bruchova H Merkerova M Prchal J. Aberrant expression of microRNA in polycythemia vera. *Haematologica.* (2008) 93:1009–16. 10.3324/haematol.12706 18508790

[35] Bray P McKenzie S Edelstein L Nagalla S Delgrosso K Ertel A The complex transcriptional landscape of the anucleate human platelet. *BMC Genomics.* (2013) 14:1. 10.1186/1471-2164-14-1 23323973 PMC3722126

[36] Krammer T Mayr M Hackl M. microRNAs as promising biomarkers of platelet activity in antiplatelet therapy monitoring. *Int J Mol Sci.* (2020) 21:3477. 10.3390/ijms21103477 32423125 PMC7278969

[37] Simon L Edelstein L Nagalla S Woodley A Chen E Kong X Human platelet microRNA-mRNA networks associated with age and gender revealed by integrated plateletomics. *Blood.* (2014) 123:e37–45. 10.1182/blood-2013-12-544692 24523238 PMC3990915

[38] Osman A Fälker K. Characterization of human platelet microRNA by quantitative PCR coupled with an annotation network for predicted target genes. *Platelets.* (2011) 22:433–41. 10.3109/09537104.2011.560305 21438667

[39] Ambrose A Alsahli M Kurmani S Goodall A. Comparison of the release of microRNAs and extracellular vesicles from platelets in response to different agonists. *Platelets.* (2018) 29:446–54. 10.1080/09537104.2017.1332366 28727490

[40] Potts K Farley A Dawson C Rimes J Biben C de Graaf C Membrane budding is a major mechanism of in vivo platelet biogenesis. *J Exp Med.* (2020) 217:e20191206. 10.1084/jem.20191206 32706855 PMC7478734

[41] Grozovsky R Giannini S Falet H Hoffmeister K. Regulating billions of blood platelets: glycans and beyond. *Blood.* (2015) 126:1877–84. 10.1182/blood-2015-01-569129 26330242 PMC4608239

[42] Gatsiou A Boeckel J Randriamboavonjy V Stellos K. MicroRNAs in platelet biogenesis and function: implications in vascular homeostasis and inflammation. *Curr Vasc Pharmacol.* (2012) 10:524–31. 10.2174/157016112801784611 22338566

[43] Garzon R Pichiorri F Palumbo T Iuliano R Cimmino A Aqeilan R MicroRNA fingerprints during human megakaryocytopoiesis. *Proc Natl Acad Sci U S A.* (2006) 103:5078–83. 10.1073/pnas.0600587103 16549775 PMC1458797

[44] Navarro F Gutman D Meire E Cáceres M Rigoutsos I Bentwich Z miR-34a contributes to megakaryocytic differentiation of K562 cells independently of p53. *Blood.* (2009) 114:2181–92. 10.1182/blood-2009-02-205062 19584398

[45] Lu J Guo S Ebert B Zhang H Peng X Bosco J MicroRNA-mediated control of cell fate in megakaryocyte-erythrocyte progenitors. *Dev Cell.* (2008) 14:843–53. 10.1016/j.devcel.2008.03.012 18539114 PMC2688789

[46] Girardot M Pecquet C Boukour S Knoops L Ferrant A Vainchenker W miR-28 is a thrombopoietin receptor targeting microRNA detected in a fraction of myeloproliferative neoplasm patient platelets. *Blood.* (2010) 116:437–45. 10.1182/blood-2008-06-165985 20445018

[47] Zhang Z Ran Y Shaw T Peng Y. MicroRNAs 10a and 10b regulate the expression of human platelet glycoprotein ibα for normal megakaryopoiesis. *Int J Mol Sci.* (2016) 17:1873. 10.3390/ijms17111873 27834869 PMC5133873

[48] Romania P Lulli V Pelosi E Biffoni M Peschle C Marziali G. MicroRNA 155 modulates megakaryopoiesis at progenitor and precursor level by targeting Ets-1 and Meis1 transcription factors. *Br J Haematol.* (2008) 143:570–80. 10.1111/j.1365-2141.2008.07382.x 18950466

[49] Bhatlekar S Manne B Basak I Edelstein L Tugolukova E Stoller M miR-125a-5p regulates megakaryocyte proplatelet formation via the actin-bundling protein L-plastin. *Blood.* (2020) 136:1760–72. 10.1182/blood.2020005230 32844999 PMC7544541

[50] Baj-Krzyworzeka M Majka M Pratico D Ratajczak J Vilaire G Kijowski J Platelet-derived microparticles stimulate proliferation, survival, adhesion, and chemotaxis of hematopoietic cells. *Exp Hematol.* (2002) 30:450–9. 10.1016/s0301-472x(02)00791-9 12031651

[51] Janowska-Wieczorek A Majka M Kijowski J Baj-Krzyworzeka M Reca R Turner A Platelet-derived microparticles bind to hematopoietic stem/progenitor cells and enhance their engraftment. *Blood.* (2001) 98:3143–9. 10.1182/blood.v98.10.3143 11698303

[52] Yuan J Wang F Yu J Yang G Liu X Zhang J. MicroRNA-223 reversibly regulates erythroid and megakaryocytic differentiation of K562 cells. *J Cell Mol Med.* (2009) 13:4551–9. 10.1111/j.1582-4934.2008.00585.x 19017354 PMC4515070

[53] Leierseder S Petzold T Zhang L Loyer X Massberg S Engelhardt S. MiR-223 is dispensable for platelet production and function in mice. *Thromb Haemost.* (2013) 110:1207–14. 10.1160/TH13-07-0623 24067995

[54] Qu M Zou X Fang F Wang S Xu L Zeng Q Platelet-derived microparticles enhance megakaryocyte differentiation and platelet generation via miR-1915-3p. *Nat Commun.* (2020) 11:4964. 10.1038/s41467-020-18802-0 33009394 PMC7532443

[55] Long M Williams N Ebbe S. Immature megakaryocytes in the mouse: physical characteristics, cell cycle status, and in vitro responsiveness to thrombopoietic stimulatory factor. *Blood.* (1982) 59:569–75. 7059669

[56] Kaushansky A Kaushansky K. Systems biology of megakaryocytes. *Adv Exp Med Biol.* (2014) 844:59–84. 10.1007/978-1-4939-2095-2_4 25480637

[57] Vitrat N Cohen-Solal K Pique C Le Couedic J Norol F Larsen A Endomitosis of human megakaryocytes are due to abortive mitosis. *Blood.* (1998) 91:3711–23. 9573008

[58] Odell T Jackson C Friday T. Megakaryocytopoiesis in rats with special reference to polyploidy. *Blood.* (1970) 35:775–82. 5427247

[59] Nakao K Angrist A. Membrane surface specialization of blood platelet and megakaryocyte. *Nature.* (1968) 217:960–1. 10.1038/217960a0 4230624

[60] Radley J Haller C. The demarcation membrane system of the megakaryocyte: a misnomer? *Blood.* (1982) 60:213–9. 7082839

[61] Wang Y Niu Z Guo Y Wang L Lin F Zhang J. IL-11 promotes the treatment efficacy of hematopoietic stem cell transplant therapy in aplastic anemia model mice through a NF-κB/microRNA-204/thrombopoietin regulatory axis. *Exp Mol Med.* (2017) 49:e410. 10.1038/emm.2017.217 29217821 PMC5750475

[62] Kaushansky K. Thrombopoietin: a tool for understanding thrombopoiesis. *J Thromb Haemost.* (2003) 1:1587–92. 10.1046/j.1538-7836.2003.00273.x 12871295

[63] Chapnik E Rivkin N Mildner A Beck G Pasvolsky R Metzl-Raz E miR-142 orchestrates a network of actin cytoskeleton regulators during megakaryopoiesis. *Elife.* (2014) 3:e01964. 10.7554/eLife.01964 24859754 PMC4067751

[64] Emmrich S Henke K Hegermann J Ochs M Reinhardt D Klusmann J. miRNAs can increase the efficiency of ex vivo platelet generation. *Ann Hematol.* (2012) 91:1673–84. 10.1007/s00277-012-1517-z 22763947

[65] Amelirad A Shamsasenjan K Akbarzadehlaleh P Pashoutan Sarvar D. Signaling pathways of receptors involved in platelet activation and shedding of these receptors in stored platelets. *Adv Pharm Bull.* (2019) 9:38–47. 10.15171/apb.2019.005 31011556 PMC6468227

[66] Cimmino G Golino P. Platelet biology and receptor pathways. *J Cardiovasc Transl Res.* (2013) 6:299–309. 10.1007/s12265-012-9445-9 23307175

[67] Elgheznawy A Shi L Hu J Wittig I Laban H Pircher J Dicer cleavage by calpain determines platelet microRNA levels and function in diabetes. *Circ Res.* (2015) 117:157–65. 10.1161/CIRCRESAHA.117.305784 25944670

[68] Garcia A Dunoyer-Geindre S Zapilko V Nolli S Reny JL Fontana P. Functional validation of microRNA-126-3p as a platelet reactivity regulator using human haematopoietic stem cells. *Thromb Haemost.* (2019) 119:254–63. 10.1055/s-0038-1676802 30602197

[69] Kaudewitz D Skroblin P Bender LH Barwari T Willeit P Pechlaner R Association of MicroRNAs and YRNAs with platelet function. *Circ Res.* (2016) 118:420–32. 10.1161/CIRCRESAHA.114.305663 26646931 PMC5065093

[70] Szilágyi B Fejes Z Póliska S Pócsi M Czimmerer Z Patsalos A Reduced miR-26b expression in megakaryocytes and platelets contributes to elevated level of platelet activation status in sepsis. *Int J Mol Sci.* (2020) 21:866. 10.3390/ijms21030866 32013235 PMC7036890

[71] Dahiya N Atreya CD. MiR-181a reduces platelet activation via the inhibition of endogenous RAP1B. *Microrna.* (2020) 9:240–6. 10.2174/2211536608666191026120515 31738148 PMC7366005

[72] Nagalla S Shaw C Kong X Kondkar AA Edelstein LC Ma L Platelet microRNA-mRNA coexpression profiles correlate with platelet reactivity. *Blood.* (2011) 117:5189–97. 10.1182/blood-2010-09-299719 21415270 PMC3109541

[73] Blair P Flaumenhaft R. Platelet alpha-granules: basic biology and clinical correlates. *Blood Rev.* (2009) 23:177–89. 10.1016/j.blre.2009.04.001 19450911 PMC2720568

[74] Heijnen HF Schiel AE Fijnheer R Geuze HJ Sixma JJ. Activated platelets release two types of membrane vesicles: microvesicles by surface shedding and exosomes derived from exocytosis of multivesicular bodies and alpha-granules. *Blood.* (1999) 94:3791–9. 10572093

[75] Dupuis A Bordet JC Eckly A Gachet C. Platelet δ-storage pool disease: an update. *J Clin Med.* (2020) 9:2508. 10.3390/jcm9082508 32759727 PMC7466064

[76] King SM Reed GL. Development of platelet secretory granules. *Semin Cell Dev Biol.* (2002) 13:293–302. 10.1016/s1084952102000599 12243729

[77] Rubenstein DA Yin W. Platelet-activation mechanisms and vascular remodeling. *Compr Physiol.* (2018) 8:1117–56. 10.1002/cphy.c170049 29978900

[78] Südhof TC Rothman JE. Membrane fusion: grappling with SNARE and SM proteins. *Science.* (2009) 323:474–7. 10.1126/science.1161748 19164740 PMC3736821

[79] Flaumenhaft R. Molecular basis of platelet granule secretion. *Arterioscler Thromb Vasc Biol.* (2003) 23:1152–60. 12738684 10.1161/01.ATV.0000075965.88456.48

[80] Rendu F Brohard-Bohn B. The platelet release reaction: granules’ constituents, secretion and functions. *Platelets.* (2001) 12:261–73. 10.1080/09537100120068170 11487378

[81] Ponomareva AA Nevzorova TA Mordakhanova ER Andrianova IA Rauova L Litvinov RI Intracellular origin and ultrastructure of platelet-derived microparticles. *J Thromb Haemost.* (2017) 15:1655–67. 10.1111/jth.13745 28561434 PMC5657319

[82] Holinstat M. Normal platelet function. *Cancer Metastasis Rev.* (2017) 36:195–8. 28667366 10.1007/s10555-017-9677-x PMC5709181

[83] Söllner T Whiteheart SW Brunner M Erdjument-Bromage H Geromanos S Tempst P SNAP receptors implicated in vesicle targeting and fusion. *Nature.* (1993) 362:318–24. 10.1038/362318a0 8455717

[84] Edelstein LC Simon LM Montoya RT Holinstat M Chen ES Bergeron A Racial differences in human platelet PAR4 reactivity reflect expression of PCTP and miR-376c. *Nat Med.* (2013) 19:1609–16. 10.1038/nm.3385 24216752 PMC3855898

[85] Zaldivia MTK McFadyen JD Lim B Wang X Peter K. Platelet-derived microvesicles in cardiovascular diseases. *Front Cardiovasc Med.* (2017) 4:74. 10.3389/fcvm.2017.00074 29209618 PMC5702324

[86] Laffont B Corduan A Plé H Duchez AC Cloutier N Boilard E Activated platelets can deliver mRNA regulatory Ago2microRNA complexes to endothelial cells via microparticles. *Blood.* (2013) 122:253–61. 10.1182/blood-2013-03-492801 23652806

[87] Diehl P Fricke A Sander L Stamm J Bassler N Htun N Microparticles: major transport vehicles for distinct microRNAs in circulation. *Cardiovasc Res.* (2012) 93:633–44. 10.1093/cvr/cvs007 22258631 PMC3291092

[88] Chevillet JR Kang Q Ruf IK Briggs HA Vojtech LN Hughes SM Quantitative and stoichiometric analysis of the microRNA content of exosomes. *Proc Natl Acad Sci U S A.* (2014) 111:14888–93. 25267620 10.1073/pnas.1408301111 PMC4205618

[89] Provost P. The clinical significance of platelet microparticle-associated microRNAs. *Clin Chem Lab Med.* (2017) 55:657–66. 10.1515/cclm-2016-0895 28099120

[90] Berckmans RJ Nieuwland R Böing AN Romijn FP Hack CE Sturk A. Cell-derived microparticles circulate in healthy humans and support low grade thrombin generation. *Thromb Haemost.* (2001) 85:639–46. 11341498

[91] Willeit P Zampetaki A Dudek K Kaudewitz D King A Kirkby NS Circulating microRNAs as novel biomarkers for platelet activation. *Circ Res.* (2013) 112:595–600. 10.1161/CIRCRESAHA.111.300539 23283721

[92] Takeuchi K Satoh M Kuno H Yoshida T Kondo H Takeuchi M. Platelet-like particle formation in the human megakaryoblastic leukaemia cell lines, MEG-01 and MEG-01s. *Br J Haematol.* (1998) 100:436–44. 10.1046/j.1365-2141.1998.00576.x 9488640

[93] Pan Y Liang H Liu H Li D Chen X Li L Platelet-secreted microRNA-223 promotes endothelial cell apoptosis induced by advanced glycation end products via targeting the insulin-like growth factor 1 receptor. *J Immunol.* (2014) 192:437–46. 10.4049/jimmunol.1301790 24307738

[94] Li J Tan M Xiang Q Zhou Z Yan H. Thrombin-activated platelet-derived exosomes regulate endothelial cell expression of ICAM-1 via microRNA-223 during the thrombosis-inflammation response. *Thromb Res.* (2017) 154:96–105. 10.1016/j.thromres.2017.04.016 28460288

[95] Zhang Y Zhang W Zha C Liu Y. Platelets activated by the anti-β2GPI/β2GPI complex release microRNAs to inhibit migration and tube formation of human umbilical vein endothelial cells. *Cell Mol Biol Lett.* (2018) 23:24. 10.1186/s11658-018-0091-3 29785186 PMC5952642

[96] Anene C Graham AM Boyne J Roberts W. Platelet microparticle delivered microRNA-Let-7a promotes the angiogenic switch. *Biochim Biophys Acta Mol Basis Dis.* (2018) 1864:2633–43. 10.1016/j.bbadis.2018.04.013 29684582

[97] Bao H Chen YX Huang K Zhuang F Bao M Han Y Platelet-derived microparticles promote endothelial cell proliferation in hypertension via miR-142-3p. *FASEB J.* (2018) 32:3912–23. 10.1096/fj.201701073R 29481306

[98] Tan M Yan HB Li JN Li WK Fu YY Chen W Thrombin stimulated platelet-derived exosomes inhibit platelet-derived growth factor receptor-beta expression in vascular smooth muscle cells. *Cell Physiol Biochem.* (2016) 38:2348–65. 10.1159/000445588 27198239

[99] Laffont B Corduan A Rousseau M Duchez AC Lee CH Boilard E Platelet microparticles reprogram macrophage gene expression and function. *Thromb Haemost.* (2016) 115:311–23. 10.1160/TH15-05-0389 26333874

[100] Yang Y Luo H Liu S Zhang R Zhu X Liu M Platelet microparticles-containing miR-4306 inhibits human monocyte-derived macrophages migration through VEGFA/ERK1/2/NF-κB signaling pathways. *Clin Exp Hypertens.* (2019) 41:481–91. 30183452 10.1080/10641963.2018.1510941

[101] Sadallah S Schmied L Eken C Charoudeh HN Amicarella F Schifferli JA. Platelet-derived ectosomes reduce NK cell function. *J Immunol.* (2016) 197:1663–71. 10.4049/jimmunol.1502658 27448586

[102] Lazar S Goldfinger LE. Platelet microparticles and miRNA transfer in cancer progression: many targets, modes of action, and effects across cancer stages. *Front Cardiovasc Med.* (2018) 5:13. 10.3389/fcvm.2018.00013 29564336 PMC5850852

[103] Liang H Yan X Pan Y Wang Y Wang N Li L MicroRNA-223 delivered by platelet-derived microvesicles promotes lung cancer cell invasion via targeting tumor suppressor EPB41L3. *Mol Cancer.* (2015) 14:58. 10.1186/s12943-015-0327-z 25881295 PMC4360939

[104] Michael JV Wurtzel JGT Mao GF Rao AK Kolpakov MA Sabri A Platelet microparticles infiltrating solid tumors transfer miRNAs that suppress tumor growth. *Blood.* (2017) 130:567–80. 10.1182/blood-2016-11-751099 28500171 PMC5542851

[105] Fuentes E Palomo I Alarcón M. Platelet miRNAs and cardiovascular diseases. *Life Sci.* (2015) 133:29–44. 10.1016/j.lfs.2015.04.016 26003375

[106] Renga B Scavizzi F. Platelets and cardiovascular risk. *Acta Cardiol.* (2017) 72:2–8. 10.1080/00015385.2017.1281560 28597734

[107] Gay LJ Felding-Habermann B. Contribution of platelets to tumour metastasis. *Nat Rev Cancer.* (2011) 11:123–34. 10.1038/nrc3004 21258396 PMC6894505

[108] von Hundelshausen P Weber C. Platelets as immune cells: bridging inflammation and cardiovascular disease. *Circ Res.* (2007) 100:27–40. 10.1161/01.RES.0000252802.25497.b7 17204662

[109] Haemmerle M Stone RL Menter DG Afshar-Kharghan V Sood AK. The platelet lifeline to cancer: challenges and opportunities. *Cancer Cell.* (2018) 33:965–83. 10.1016/j.ccell.2018.03.002 29657130 PMC5997503

[110] Herrington W Lacey B Sherliker P Armitage J Lewington S. Epidemiology of atherosclerosis and the potential to reduce the global burden of atherothrombotic disease. *Circ Res.* (2016) 118:535–46. 10.1161/CIRCRESAHA.115.307611 26892956

[111] Gimbrone MA Jr García-Cardeña G. Endothelial cell dysfunction and the pathobiology of atherosclerosis. *Circ Res.* (2016) 118:620–36. 10.1161/CIRCRESAHA.115.306301 26892962 PMC4762052

[112] Falk E. Pathogenesis of atherosclerosis. *J Am Coll Cardiol.* (2006) 47:C7–12. 16631513 10.1016/j.jacc.2005.09.068

[113] Wolf D Ley K. Immunity and inflammation in atherosclerosis. *Circ Res.* (2019) 124:315–27. 30653442 10.1161/CIRCRESAHA.118.313591 PMC6342482

[114] Bakogiannis C Sachse M Stamatelopoulos K Stellos K. Platelet-derived chemokines in inflammation and atherosclerosis. *Cytokine.* (2019) 122:154157. 10.1016/j.cyto.2017.09.013 29198385

[115] McManus DD Freedman JE. MicroRNAs in platelet function and cardiovascular disease. *Nat Rev Cardiol.* (2015) 12:711–7. 10.1038/nrcardio.2015.101 26149483

[116] Wang Y Xie Y Zhang A Wang M Fang Z Zhang J. Exosomes: an emerging factor in atherosclerosis. *Biomed Pharmacother.* (2019) 115:108951. 10.1016/j.biopha.2019.108951 31078042

[117] Sondermeijer BM Bakker A Halliani A de Ronde MW Marquart AA Tijsen AJ Platelets in patients with premature coronary artery disease exhibit upregulation of miRNA340* and miRNA624*. *PLoS One.* (2011) 6:e25946. 10.1371/journal.pone.0025946 22022480 PMC3192762

[118] Tian HS Zhou QG Shao F. Relationship between arterial atheromatous plaque morphology and platelet-associated miR-126 and miR-223 expressions. *Asian Pac J Trop Med.* (2015) 8:309–14. 10.1016/S1995-7645(14)60336-9 25975504

[119] Hao XZ Fan HM. Identification of miRNAs as atherosclerosis biomarkers and functional role of miR-126 in atherosclerosis progression through MAPK signalling pathway. *Eur Rev Med Pharm Sci.* (2017) 21:2725–33. 28678312

[120] Alexandru N Constantin A Nemecz M Comariţa IK Vîlcu A Procopciuc A Hypertension associated with hyperlipidemia induced different microrna expression profiles in plasma, platelets, and platelet-derived microvesicles; effects of endothelial progenitor cell therapy. *Front Med.* (2019) 6:280. 10.3389/fmed.2019.00280 31850358 PMC6901790

[121] Gatsiou A Georgiopoulos G Vlachogiannis NI Pfisterer L Fischer A Sachse M Additive contribution of microRNA-34a/b/c to human arterial ageing and atherosclerosis. *Atherosclerosis.* (2021) 327:49–58. 10.1016/j.atherosclerosis.2021.05.005 34038763

[122] Su F Shi M Zhang J Zheng Q Wang H Li X MiR-223/NFAT5 signaling suppresses arterial smooth muscle cell proliferation and motility in vitro. *Aging.* (2020) 12:26188–98. 10.18632/aging.202395 33373321 PMC7803580

[123] Yao Y Sun W Sun Q Jing B Liu S Liu X Platelet-derived exosomal microRNA-25-3p inhibits coronary vascular endothelial cell inflammation through adam10 via the NF-κB signaling pathway in ApoE −/− mice. *Front Immunol.* (2019) 10:2205. 10.3389/fimmu.2019.02205 31632389 PMC6783608

[124] Hromadka M Motovska Z Hlinomaz O Kala P Tousek F Jarkovsky J MiR-126-3p and MiR-223-3p as biomarkers for prediction of thrombotic risk in patients with acute myocardial infarction and primary angioplasty. *J Pers Med.* (2021) 11:508. 10.3390/jpm11060508 34199723 PMC8230013

[125] Qiu H Zhang Y Zhao Q Jiang H Yan J Liu Y. Platelet miR-587 may be used as a potential biomarker for diagnosis of patients with acute coronary syndrome. *Clin Lab.* (2020) 66:66. 10.7754/Clin.Lab.2019.190703 32162877

[126] Zhang DM Deng JJ Wu YG Tang T Xiong L Zheng Y MicroRNA-223-3p protect against radiation-induced cardiac toxicity by alleviating myocardial oxidative stress and programmed cell death via targeting the AMPK pathway. *Front Cell Dev Biol.* (2021) 9:801661. 10.3389/fcell.2021.801661 35111759 PMC8801819

[127] Liu X Xu Y Deng Y Li H. MicroRNA-223 regulates cardiac fibrosis after myocardial infarction by targeting RASA1. *Cell Physiol Biochem.* (2018) 46:1439–54. 10.1159/000489185 29689569

[128] Liu X Deng Y Xu Y Jin W Li H. MicroRNA-223 protects neonatal rat cardiomyocytes and H9c2 cells from hypoxia-induced apoptosis and excessive autophagy via the Akt/mTOR pathway by targeting PARP-1. *J Mol Cell Cardiol.* (2018) 118:133–46. 10.1016/j.yjmcc.2018.03.018 29608885

[129] Duan S Wang C Xu X Zhang X Su G Li Y Peripheral serum exosomes isolated from patients with acute myocardial infarction promote endothelial cell angiogenesis via the miR-126-3p/TSC1/mTORC1/HIF-1α pathway. *Int J Nanomed.* (2022) 17:1577–92. 10.2147/IJN.S338937 35400999 PMC8988947

[130] Kearney PM Whelton M Reynolds K Muntner P Whelton PK He J. Global burden of hypertension: analysis of worldwide data. *Lancet.* (2005) 365:217–23. 10.1016/S0140-6736(05)17741-1 15652604

[131] Marketou M Kontaraki J Papadakis J Kochiadakis G Vrentzos G Maragkoudakis S Platelet microRNAs in hypertensive patients with and without cardiovascular disease. *J Hum Hypertens.* (2019) 33:149–56. 10.1038/s41371-018-0123-5 30375479

[132] Huang YQ Huang C Chen JY Li J Feng YQ. Plasma expression level of miRNA let-7 is positively correlated with carotid intima-media thickness in patients with essential hypertension. *J Hum Hypertens.* (2017) 31:843–7. 10.1038/jhh.2017.52 28816229

[133] Parthenakis F Marketou M Kontaraki J Patrianakos A Nakou H Touloupaki M Low levels of MicroRNA-21 are a marker of reduced arterial stiffness in well-controlled hypertension. *J Clin Hypertens.* (2017) 19:235–40. 10.1111/jch.12900 27550546 PMC8031006

[134] Fernandes T Magalhães FC Roque FR Phillips MI Oliveira EM. Exercise training prevents the microvascular rarefaction in hypertension balancing angiogenic and apoptotic factors: role of microRNAs-16, −21, and −126. *Hypertension.* (2012) 59:513–20. 10.1161/HYPERTENSIONAHA.111.185801 22215713

[135] Stamler J Vaccaro O Neaton JD Wentworth D. Diabetes, other risk factors, and 12-yr cardiovascular mortality for men screened in the Multiple Risk Factor Intervention Trial. *Diabetes Care.* (1993) 16:434–44. 10.2337/diacare.16.2.434 8432214

[136] Engelgau MM Geiss LS Saaddine JB Boyle JP Benjamin SM Gregg EW The evolving diabetes burden in the United States. *Ann Intern Med.* (2004) 140:945–50. 10.7326/0003-4819-140-11-200406010-00035 15172919

[137] Nakagami H Kaneda Y Ogihara T Morishita R. Endothelial dysfunction in hyperglycemia as a trigger of atherosclerosis. *Curr Diabetes Rev.* (2005) 1:59–63. 10.2174/1573399052952550 18220582

[138] Alberti G Zimmet P Shaw J Bloomgarden Z Kaufman F Silink M. Type 2 diabetes in the young: the evolving epidemic: the international diabetes federation consensus workshop. *Diabetes Care.* (2004) 27:1798–811. 10.2337/diacare.27.7.1798 15220270

[139] Russo I Penna C Musso T Popara J Alloatti G Cavalot F Platelets, diabetes and myocardial ischemia/reperfusion injury. *Cardiovasc Diabetol.* (2017) 16:71. 10.1186/s12933-017-0550-6 28569217 PMC5452354

[140] Nomura S Miyazaki Y Miyake T Suzuki M Kawakatsu T Kido H Detection of platelet-derived microparticles in patients with diabetes. *Am J Hematol.* (1993) 44:213. 10.1002/ajh.2830440319 8213778

[141] Liang Y Wang M Wang C Liu Y Naruse K Takahashi K. The mechanisms of the development of atherosclerosis in prediabetes. *Int J Mol Sci.* (2021) 22:4108. 10.3390/ijms22084108 33921168 PMC8071517

[142] Natarajan A Zaman AG Marshall SM. Platelet hyperactivity in type 2 diabetes: role of antiplatelet agents. *Diabetes Vasc Dis Res.* (2008) 5:138–44. 10.3132/dvdr.2008.023 18537103

[143] Ferroni P Basili S Falco A Davì G. Platelet activation in type 2 diabetes mellitus. *J Thromb Haemost.* (2004) 2:1282–91. 15304032 10.1111/j.1538-7836.2004.00836.x

[144] Fejes Z Póliska S Czimmerer Z Káplár M Penyige A Gál Szabó G Hyperglycaemia suppresses microRNA expression in platelets to increase P2RY12 and SELP levels in type 2 diabetes mellitus. *Thromb Haemost.* (2017) 117:529–42. 10.1160/TH16-04-0322 27975100

[145] Zampetaki A Mayr M. Sweet dicer: impairment of micro-RNA processing by diabetes. *Circ Res.* (2015) 117:116–8. 10.1161/CIRCRESAHA.117.306817 26139856

[146] Zhou M Gao M Luo Y Gui R Ji H. Long non-coding RNA metallothionein 1 pseudogene 3 promotes p2y12 expression by sponging miR-126 to activate platelet in diabetic animal model. *Platelets.* (2019) 30:452–9. 10.1080/09537104.2018.1457781 29617185

[147] Duan X Zhan Q Song B Zeng S Zhou J Long Y Detection of platelet microRNA expression in patients with diabetes mellitus with or without ischemic stroke. *J Diabetes Complications.* (2014) 28:705–10. 10.1016/j.jdiacomp.2014.04.012 24908639

[148] Luo M Li R Deng X Ren M Chen N Zeng M Platelet-derived miR-103b as a novel biomarker for the early diagnosis of type 2 diabetes. *Acta Diabetol.* (2015) 52:943–9. 10.1007/s00592-015-0733-0 25820527

[149] Luo M Li R Ren M Chen N Deng X Tan X Hyperglycaemia-induced reciprocal changes in miR-30c and PAI-1 expression in platelets. *Sci Rep.* (2016) 6:36687. 10.1038/srep36687 27819307 PMC5098184

[150] Prattichizzo F Giuliani A De Nigris V Pujadas G Ceka A La Sala L Extracellular microRNAs and endothelial hyperglycaemic memory: a therapeutic opportunity? *Diabetes Obes Metab.* (2016) 18:855–67. 10.1111/dom.12688 27161301 PMC5094499

[151] Xue WL Chen RQ Zhang QQ Li XH Cao L Li MY Hydrogen sulfide rescues high glucose-induced migration dysfunction in HUVECs by upregulating miR-126-3p. *Am J Physiol Cell Physiol.* (2020) 318:C857–69. 10.1152/ajpcell.00406.2019 32186933

[152] Cavarretta E Chiariello GA Condorelli G. Platelets, endothelium, and circulating microRNA-126 as a prognostic biomarker in cardiovascular diseases: per aspirin ad astra. *Eur Heart J.* (2013) 34:3400–2. 10.1093/eurheartj/eht032 23391580

[153] Shao M Yu M Zhao J Mei J Pan Y Zhang J miR-21-3p regulates AGE/RAGE signalling and improves diabetic atherosclerosis. *Cell Biochem Funct.* (2020) 38:965–75. 10.1002/cbf.3523 32196704

[154] Deng B Hu Y Sheng X Zeng H Huo Y. miR-223-3p reduces high glucose and high fat-induced endothelial cell injury in diabetic mice by regulating NLRP3 expression. *Exp Ther Med.* (2020) 20:1514–20. 10.3892/etm.2020.8864 32765674 PMC7388564

[155] Brennan E Wang B McClelland A Mohan M Marai M Beuscart O Protective effect of let-7 miRNA family in regulating inflammation in diabetes-associated atherosclerosis. *Diabetes.* (2017) 66:2266–77. 10.2337/db16-1405 28487436

[156] Akbarinia A Kargarfard M Naderi M. Aerobic training improves platelet function in type 2 diabetic patients: role of microRNA-130a and GPIIb. *Acta Diabetol.* (2018) 55:893–9. 10.1007/s00592-018-1167-2 29855803

[157] Taghizadeh M Kargarfard M Braune S Jung F Naderi M. Long-term aerobic exercise training in type two diabetic patients alters the expression of miRNA-223 and its corresponding target, the P2RY12 receptor, attenuating platelet function. *Clin Hemorheol Microcirc.* (2021) 80:107–16. 34420942 10.3233/CH-211209

[158] Taghizadeh M Ahmadizad S Naderi M. Effects of endurance training on hsa-miR-223, P2RY12 receptor expression and platelet function in type 2 diabetic patients. *Clin Hemorheol Microcirc.* (2018) 68:391–9. 10.3233/CH-170300 29526844

[159] de Boer HC van Solingen C Prins J Duijs JM Huisman MV Rabelink TJ Aspirin treatment hampers the use of plasma microRNA-126 as a biomarker for the progression of vascular disease. *Eur Heart J.* (2013) 34:3451–7. 10.1093/eurheartj/eht007 23386708

[160] Sabatino J De Rosa S Polimeni A Sorrentino S Indolfi C. Direct oral anticoagulants in patients with active cancer: a systematic review and meta-analysis. *JACC Cardiooncol.* (2020) 2:428–40. 34396250 10.1016/j.jaccao.2020.06.001 PMC8352218

[161] Camilli M Iannaccone G La Vecchia G Cappannoli L Scacciavillani R Minotti G Platelets: the point of interconnection among cancer, inflammation and cardiovascular diseases. *Expert Rev Hematol.* (2021) 14:537–46. 10.1080/17474086.2021.1943353 34126832

[162] Baron JA Cole BF Sandler RS Haile RW Ahnen D Bresalier R A randomized trial of aspirin to prevent colorectal adenomas. *N Engl J Med.* (2003) 348:891–9. 12621133 10.1056/NEJMoa021735

[163] Rothwell PM Wilson M Elwin CE Norrving B Algra A Warlow CP Long-term effect of aspirin on colorectal cancer incidence and mortality: 20-year follow-up of five randomised trials. *Lancet.* (2010) 376:1741–50. 10.1016/S0140-6736(10)61543-7 20970847

[164] Beavers CJ Rodgers JE Bagnola AJ Beckie TM Campia U Di Palo KE Cardio-oncology drug interactions: a scientific statement from the American heart association. *Circulation.* (2022) 145:e811–38. 10.1161/CIR.0000000000001056 35249373

[165] Plantureux L Mège D Crescence L Carminita E Robert S Cointe S The interaction of platelets with colorectal cancer cells inhibits tumor growth but promotes metastasis. *Cancer Res.* (2020) 80:291–303. 10.1158/0008-5472.CAN-19-1181 31727628

[166] Weinstein MP. Comparative in vitro activity of lomefloxacin and other antimicrobials against 597 microorganisms causing bacteremia. *Diagn Microbiol Infect Dis.* (1988) 11:195–200. 10.1016/0732-8893(88)90003-x 3071448

[167] Wurtzel JGT Lazar S Sikder S Cai KQ Astsaturov I Weyrich AS Platelet microRNAs inhibit primary tumor growth via broad modulation of tumor cell mRNA expression in ectopic pancreatic cancer in mice. *PLoS One.* (2021) 16:e0261633. 10.1371/journal.pone.0261633 34936674 PMC8694476

[168] Yang M Chen J Su F Yu B Su F Lin L Microvesicles secreted by macrophages shuttle invasion-potentiating microRNAs into breast cancer cells. *Mol Cancer.* (2011) 10:117. 10.1186/1476-4598-10-117 21939504 PMC3190352

[169] Hu Y Yi B Chen X Xu L Zhou X Zhu X. MiR-223 promotes tumor progression via targeting rhob in gastric cancer. *J Oncol.* (2022) 2022:6708871. 10.1155/2022/6708871 35035482 PMC8758265

[170] Li X Zhang Y Zhang H Liu X Gong T Li M miRNA-223 promotes gastric cancer invasion and metastasis by targeting tumor suppressor EPB41L3. *Mol Cancer Res.* (2011) 9:824–33. 21628394 10.1158/1541-7786.MCR-10-0529

[171] Gasperi V Vangapandu C Savini I Ventimiglia G Adorno G Catani MV. Polyunsaturated fatty acids modulate the delivery of platelet microvesicle-derived microRNAs into human breast cancer cell lines. *J Nutr Biochem.* (2019) 74:108242. 10.1016/j.jnutbio.2019.108242 31665654

[172] Liu L Yuan L Huang D Han Q Cai J Wang S miR-126 regulates the progression of epithelial ovarian cancer in vitro and in vivo by targeting VEGF-A. *Int J Oncol.* (2020) 57:825–34. 10.3892/ijo.2020.5082 32705156

[173] Xu J Wang H Wang H Chen Q Zhang L Song C The inhibition of miR-126 in cell migration and invasion of cervical cancer through regulating ZEB1. *Hereditas.* (2019) 156:11. 10.1186/s41065-019-0087-7 31007650 PMC6456986

[174] Hua Y Liang C Miao C Wang S Su S Shao P MicroRNA-126 inhibits proliferation and metastasis in prostate cancer via regulation of ADAM9. *Oncol Lett.* (2018) 15:9051–60. 10.3892/ol.2018.8528 29805636 PMC5958673

[175] Liu R Zhang YS Zhang S Cheng ZM Yu JL Zhou S MiR-126-3p suppresses the growth, migration and invasion of NSCLC via targeting CCR1. *Eur Rev Med Pharm Sci.* (2019) 23:679–89. 10.26355/eurrev_201901_16881 30720175

[176] Li M Meng X Li M. MiR-126 promotes esophageal squamous cell carcinoma via inhibition of apoptosis and autophagy. *Aging.* (2020) 12:12107–18. 10.18632/aging.103379 32554852 PMC7343473

[177] Ebrahimi F Gopalan V Smith RA Lam AK. miR-126 in human cancers: clinical roles and current perspectives. *Exp Mol Pathol.* (2014) 96:98–107. 10.1016/j.yexmp.2013.12.004 24368110

[178] Tang M Jiang L Lin Y Wu X Wang K He Q Platelet microparticle-mediated transfer of miR-939 to epithelial ovarian cancer cells promotes epithelial to mesenchymal transition. *Oncotarget.* (2017) 8:97464–75. 10.18632/oncotarget.22136 29228624 PMC5722576

[179] Pan B Chen Y Song H Xu Y Wang R Chen L. Mir-24-3p downregulation contributes to VP16-DDP resistance in small-cell lung cancer by targeting ATG4A. *Oncotarget.* (2015) 6:317–31. 10.18632/oncotarget.2787 25426560 PMC4381597

[180] Liu H Huang X Ye T. MiR-22 down-regulates the proto-oncogene ATP citrate lyase to inhibit the growth and metastasis of breast cancer. *Am J Transl Res.* (2018) 10:659–69. 29636857 PMC5883108

[181] Miao X Rahman MF Jiang L Min Y Tan S Xie H Thrombin-reduced miR-27b attenuates platelet angiogenic activities in vitro via enhancing platelet synthesis of anti-angiogenic thrombospondin-1. *J Thromb Haemost.* (2018) 16:791–801. 10.1111/jth.13978 29442415

[182] He Q Ye A Ye W Liao X Qin G Xu Y Cancer-secreted exosomal miR-21-5p induces angiogenesis and vascular permeability by targeting KRIT1. *Cell Death Dis.* (2021) 12:576. 10.1038/s41419-021-03803-8 34088891 PMC8178321

[183] Liu LZ Li C Chen Q Jing Y Carpenter R Jiang Y MiR-21 induced angiogenesis through AKT and ERK activation and HIF-1α expression. *PLoS One.* (2011) 6:e19139. 10.1371/journal.pone.0019139 21544242 PMC3081346

[184] Shi L Fisslthaler B Zippel N Frömel T Hu J Elgheznawy A MicroRNA-223 antagonizes angiogenesis by targeting β1 integrin and preventing growth factor signaling in endothelial cells. *Circ Res.* (2013) 113:1320–30. 10.1161/CIRCRESAHA.113.301824 24044949

[185] Bozec A Zangari J Butori-Pepino M Ilie M Lalvee S Juhel T MiR-223-3p inhibits angiogenesis and promotes resistance to cetuximab in head and neck squamous cell carcinoma. *Oncotarget.* (2017) 8:57174–86. 10.18632/oncotarget.19170 28915663 PMC5593634

[186] Yu Q Xiang L Yin L Liu X Yang D Zhou J. Loss-of-function of miR-142 by hypermethylation promotes TGF-β-mediated tumour growth and metastasis in hepatocellular carcinoma. *Cell Prolif.* (2017) 50:e12384. 10.1111/cpr.12384 28963738 PMC6529086

[187] Gu Y Pais G Becker V Körbel C Ampofo E Ebert E Suppression of endothelial miR-22 mediates non-small cell lung cancer cell-induced angiogenesis. *Mol Ther Nucleic Acids.* (2021) 26:849–64. 10.1016/j.omtn.2021.10.003 34729252 PMC8536510

[188] Wisman PP Roest M Asselbergs FW de Groot PG Moll FL van der Graaf Y Platelet-reactivity tests identify patients at risk of secondary cardiovascular events: a systematic review and meta-analysis. *J Thromb Haemost.* (2014) 12:736–47. 10.1111/jth.12538 24612413

[189] Farré AJ Tamargo J Mateos-Cáceres PJ Azcona L Macaya C. Old and new molecular mechanisms associated with platelet resistance to antithrombotics. *Pharm Res.* (2010) 27:2365–73. 10.1007/s11095-010-0209-4 20628791

[190] Stuckey TD Kirtane AJ Brodie BR Witzenbichler B Litherland C Weisz G Impact of aspirin and clopidogrel hyporesponsiveness in patients treated with drug-eluting stents: 2-year results of a prospective, multicenter registry study. *JACC Cardiovasc Interv.* (2017) 10:1607–17. 10.1016/j.jcin.2017.05.059 28780034

[191] Buonamici P Marcucci R Migliorini A Gensini GF Santini A Paniccia R Impact of platelet reactivity after clopidogrel administration on drug-eluting stent thrombosis. *J Am Coll Cardiol.* (2007) 49:2312–7. 10.1016/j.jacc.2007.01.094 17572245

[192] Freitas RCC Bortolin RH Lopes MB Hirata MH Hirata RDC Silbiger VN Integrated analysis of miRNA and mRNA gene expression microarrays: influence on platelet reactivity, clopidogrel response and drug-induced toxicity. *Gene.* (2016) 593:172–8. 10.1016/j.gene.2016.08.028 27543010

[193] Jäger B Stojkovic S Haller PM Piackova E Kahl BS Andric T Course of platelet miRNAs after cessation of P2Y12 antagonists. *Eur J Clin Invest.* (2019) 49:e13149. 10.1111/eci.13149 31172515

[194] Braza-Boïls A Barwari T Gutmann C Thomas MR Judge HM Joshi A Circulating MicroRNA levels indicate platelet and leukocyte activation in endotoxemia despite platelet P2Y_12_ inhibition. *Int J Mol Sci.* (2020) 21:2897. 10.3390/ijms21082897 32326325 PMC7215420

[195] Liu J Qin L Wang Z Peng L Liu J Wang X Platelet-derived miRNAs as determinants of the antiplatelet response in clopidogrel-treated patients with ACS. *Thromb Res.* (2020) 186:71–4. 10.1016/j.thromres.2019.12.016 31891827

[196] Carino A De Rosa S Sorrentino S Polimeni A Sabatino J Caiazzo G Modulation of circulating MicroRNAs levels during the switch from clopidogrel to ticagrelor. *Biomed Res Int.* (2016) 2016:3968206. 10.1155/2016/3968206 27366745 PMC4913053

[197] Parker WAE Schulte C Barwari T Phoenix F Pearson SM Mayr M Aspirin, clopidogrel and prasugrel monotherapy in patients with type 2 diabetes mellitus: a double-blind randomised controlled trial of the effects on thrombotic markers and microRNA levels. *Cardiovasc Diabetol.* (2020) 19:3. 10.1186/s12933-019-0981-3 31910903 PMC6945631

[198] Chyrchel B Totoń-Żurańska J Kruszelnicka O Chyrchel M Mielecki W Kołton-Wróż M Association of plasma miR-223 and platelet reactivity in patients with coronary artery disease on dual antiplatelet therapy: a preliminary report. *Platelets.* (2015) 26:593–7. 10.3109/09537104.2014.974527 25350775

[199] Voora D Ginsburg GS Akerblom A. Platelet RNA as a novel biomarker for the response to antiplatelet therapy. *Future Cardiol.* (2014) 10:9–12. 10.2217/fca.13.90 24344654

[200] Yu XY Chen JY Zheng ZW Wu H Li LW Zhang ZW Plasma miR-126 as a potential marker predicting major adverse cardiac events in dual antiplatelet-treated patients after percutaneous coronary intervention. *EuroIntervention.* (2013) 9:546–54. 10.4244/EIJV9I5A90 24058072

[201] Li X Yao Q Cui H Yang J Wu N Liu Y MiR-223 or miR-126 predicts resistance to dual antiplatelet therapy in patients with ST-elevation myocardial infarction. *J Int Med Res.* (2021) 49:3000605211016209. 10.1177/03000605211016209 34098766 PMC8191085

[202] Kok MG Mandolini C Moerland PD de Ronde MW Sondermeijer BM Halliani A Low miR-19b-1-5p expression in isolated platelets after aspirin use is related to aspirin insensitivity. *Int J Cardiol.* (2016) 203:262–3. 10.1016/j.ijcard.2015.10.098 26519680

[203] Chen YC Lin FY Lin YW Cheng SM Chang CC Lin RH Platelet MicroRNA 365-3p expression correlates with high on-treatment platelet reactivity in coronary artery disease patients. *Cardiovasc Drugs Ther.* (2019) 33:129–37. 10.1007/s10557-019-06855-3 30783954

[204] Ding T Zeng X Cheng B Ma X Yuan H Nie X Platelets in acute coronary syndrome patients with high platelet reactivity after dual antiplatelet therapy exhibit upregulation of miR-204-5p. *Ann Clin Lab Sci.* (2019) 49:619–31. 31611205

[205] Lin S Xu X Hu H Cheng J Chen R Hu Y The expression profile of platelet-derived miRNA in coronary artery disease patients with clopidogrel resistance. *Pharm Res Perspect.* (2021) 9:e00751. 10.1002/prp2.751 33724726 PMC7962021

[206] Rytkin E Mirzaev K Bure I Akmalova K Abdullaev S Kachanova A MicroRNAs as novel biomarkers for P2Y12 – inhibitors resistance prediction. *Pharmgenomics Pers Med.* (2021) 14:1575–82. 10.2147/PGPM.S324612 34880651 PMC8648096

[207] Liu WW Wang H Chen XH Fu SW Liu ML. miR-34b-3p may promote antiplatelet efficiency of aspirin by inhibiting thromboxane synthase expression. *Thromb Haemost.* (2019) 119:1451–60. 10.1055/s-0039-1692681 31266078

[208] Zhang Q Zhu F Luo Y Liao J Cao J Xue T. Platelet miR-107 participates in clopidogrel resistance after PCI treatment by regulating P2Y12. *Acta Haematol.* (2022) 145:46–53. 10.1159/000517811 34474410

[209] Shi R Zhou X Ji WJ Zhang YY Ma YQ Zhang JQ The emerging role of miR-223 in platelet reactivity: implications in antiplatelet therapy. *Biomed Res Int.* (2015) 2015:981841. 10.1155/2015/981841 26221610 PMC4499381

[210] Wang J Yao Y Zhang J Tang X Meng X Wang M Platelet microRNA-15b protects against high platelet reactivity in patients undergoing percutaneous coronary intervention through Bcl-2-mediated platelet apoptosis. *Ann Transl Med.* (2020) 8:364. 10.21037/atm.2020.02.88 32355808 PMC7186638

[211] Yeung J Li W Holinstat M. Platelet signaling and disease: targeted therapy for thrombosis and other related diseases. *Pharmacol Rev.* (2018) 70:526–48. 10.1124/pr.117.014530 29925522 PMC6013590

[212] Cheng Y Tan N Yang J Liu X Cao X He P A translational study of circulating cell-free microRNA-1 in acute myocardial infarction. *Clin Sci (Lond).* (2010) 119:87–95. 10.1042/CS20090645 20218970 PMC3593815

[213] Grabmaier U Clauss S Gross L Klier I Franz WM Steinbeck G Diagnostic and prognostic value of miR-1 and miR-29b on adverse ventricular remodeling after acute myocardial infarction - The SITAGRAMI-miR analysis. *Int J Cardiol.* (2017) 244:30–6. 10.1016/j.ijcard.2017.06.054 28663047

[214] Pedersen OB Grove EL Kristensen SD Nissen PH Hvas AM. MicroRNA as biomarkers for platelet function and maturity in patients with cardiovascular disease. *Thromb Haemost.* (2021) 122:181–95. 10.1055/s-0041-1730375 34091883

[215] Li S Guo LZ Kim MH Han JY Serebruany V. Platelet microRNA for predicting acute myocardial infarction. *J Thromb Thrombolysis.* (2017) 44:556–64. 10.1007/s11239-017-1537-6 29030746

[216] Pordzik J Eyileten-Postuła C Jakubik D Czajka P Nowak A De Rosa S MiR-126 is an independent predictor of long-term all-cause mortality in patients with type 2 diabetes mellitus. *J Clin Med.* (2021) 10:2371. 10.3390/jcm10112371 34071189 PMC8198825

[217] Rossetti P Goldoni M Pengo V Vescovini R Mozzoni P Tassoni MI MiRNA 126 as a new predictor biomarker in venous thromboembolism of persistent residual vein obstruction: a review of the literature plus a pilot study. *Semin Thromb Hemost.* (2021) 47:982–91. 10.1055/s-0041-1726341 34243207

[218] Schulte C Molz S Appelbaum S Karakas M Ojeda F Lau DM miRNA-197 and miRNA-223 predict cardiovascular death in a cohort of patients with symptomatic coronary artery disease. *PLoS One.* (2015) 10:e0145930. 10.1371/journal.pone.0145930 26720041 PMC4699820

[219] Szelenberger R Karbownik MS Kacprzak M Maciak K Bijak M Zielińska M Screening analysis of platelet miRNA profile revealed miR-142-3p as a potential biomarker in modeling the risk of acute coronary syndrome. *Cells.* (2021) 10:3526. 10.3390/cells10123526 34944034 PMC8700136

[220] Bijak M Dzieciol M Rywaniak J Saluk J Zielinska M. Platelets miRNA as a prediction marker of thrombotic episodes. *Dis Markers.* (2016) 2016:2872507. 10.1155/2016/2872507 28042196 PMC5155104

[221] Yang S Zhao J Chen Y Lei M. Biomarkers associated with ischemic stroke in diabetes mellitus patients. *Cardiovasc Toxicol.* (2016) 16:213–22. 10.1007/s12012-015-9329-8 26175178

[222] In ’t Veld S Wurdinger T. Tumor-educated platelets. *Blood.* (2019) 133:2359–64. 30833413 10.1182/blood-2018-12-852830

[223] Vaidyanathan R Soon RH Zhang P Jiang K Lim CT. Cancer diagnosis: from tumor to liquid biopsy and beyond. *Lab Chip.* (2018) 19:11–34. 30480287 10.1039/c8lc00684a

[224] Best MG Wesseling P Wurdinger T. Tumor-educated platelets as a noninvasive biomarker source for cancer detection and progression monitoring. *Cancer Res.* (2018) 78:3407–12. 10.1158/0008-5472.CAN-18-0887 29921699

[225] Miao S Zhang Q Chang W Wang J. New insights into platelet-enriched miRNAs: production, functions, roles in tumors, and potential targets for tumor diagnosis and treatment. *Mol Cancer Ther.* (2021) 20:1359–66. 10.1158/1535-7163.MCT-21-0050 34045229

[226] Wang H Wei X Wu B Su J Tan W Yang K. Tumor-educated platelet miR-34c-3p and miR-18a-5p as potential liquid biopsy biomarkers for nasopharyngeal carcinoma diagnosis. *Cancer Manage Res.* (2019) 11:3351–60. 10.2147/CMAR.S195654 31114371 PMC6489554

[227] Best MG Sol N Kooi I Tannous J Westerman BA Rustenburg F RNA-Seq of tumor-educated platelets enables blood-based pan-cancer, multiclass, and molecular pathway cancer diagnostics. *Cancer Cell.* (2015) 28:666–76. 10.1016/j.ccell.2015.09.018 26525104 PMC4644263

[228] Zhong S Golpon H Zardo P Borlak J. miRNAs in lung cancer. A systematic review identifies predictive and prognostic miRNA candidates for precision medicine in lung cancer. *Transl Res.* (2021) 230:164–96. 10.1016/j.trsl.2020.11.012 33253979

[229] Omland T. Cardio-protective therapy in cardio-oncology: quo vadis? *Circulation.* (2021) 144:667–9. 10.1161/CIRCULATIONAHA.121.055541 34460325

[230] Delgado Lagos F Elgheznawy A Kyselova A Meyer Zu Heringdorf D Ratiu C Randriamboavonjy V Secreted modular calcium-binding protein 1 binds and activates thrombin to account for platelet hyperreactivity in diabetes. *Blood.* (2021) 137:1641–51. 10.1182/blood.2020009405 33529332

[231] Zhou Y Abraham S Andre P Edelstein LC Shaw CA Dangelmaier CA Anti-miR-148a regulates platelet FcγRIIA signaling and decreases thrombosis in vivo in mice. *Blood.* (2015) 126:2871–81. 26516227 10.1182/blood-2015-02-631135 PMC4692146

[232] Dahiya N Atreya CD. RAP1 downregulation by miR-320c reduces platelet activation in ex-vivo storage. *Microrna.* (2019) 8:36–42. 10.2174/2211536607666180521094532 29779489

[233] Kondkar AA Bray MS Leal SM Nagalla S Liu DJ Jin Y VAMP8/endobrevin is overexpressed in hyperreactive human platelets: suggested role for platelet microRNA. *J Thromb Haemost.* (2010) 8:369–78. 10.1111/j.1538-7836.2009.03700.x 19943878 PMC3312605

[234] Barwari T Eminaga S Mayr U Lu R Armstrong PC Chan MV Inhibition of profibrotic microRNA-21 affects platelets and their releasate. *JCI Insight.* (2018) 3:e123335. 10.1172/jci.insight.123335 30385722 PMC6238735

[235] Yu S Deng G Qian D Xie Z Sun H Huang D Detection of apoptosis-associated microRNA in human apheresis platelets during storage by quantitative real-time polymerase chain reaction analysis. *Blood Transfus.* (2014) 12:541–7. 10.2450/2014.0291-13 24960647 PMC4212035

[236] Huang SC Wang M Wu WB Wang R Cui J Li W Mir-22-3p inhibits arterial smooth muscle cell proliferation and migration and neointimal hyperplasia by targeting HMGB1 in arteriosclerosis obliterans. *Cell Physiol Biochem.* (2017) 42:2492–506. 10.1159/000480212 28848136

[237] Wang TM Chen KC Hsu PY Lin HF Wang YS Chen CY microRNA let-7g suppresses PDGF-induced conversion of vascular smooth muscle cell into the synthetic phenotype. *J Cell Mol Med.* (2017) 21:3592–601. 10.1111/jcmm.13269 28699690 PMC5706591

[238] Zhao XS Ren Y Wu Y Ren HK Chen H. MiR-30b-5p and miR-22-3p restrain the fibrogenesis of post-myocardial infarction in mice via targeting PTAFR. *Eur Rev Med Pharmacol Sci.* (2020) 24:3993–4004. 10.26355/eurrev_202004_20869 32329883

[239] Tang Q Li MY Su YF Fu J Zou ZY Wang Y Absence of miR-223-3p ameliorates hypoxia-induced injury through repressing cardiomyocyte apoptosis and oxidative stress by targeting KLF15. *Eur J Pharmacol.* (2018) 841:67–74. 10.1016/j.ejphar.2018.10.014 30336138

[240] Liu QQ Ren K Liu SH Li WM Huang CJ Yang XH. MicroRNA-140-5p aggravates hypertension and oxidative stress of atherosclerosis via targeting Nrf2 and Sirt2. *Int J Mol Med.* (2019) 43:839–49. 10.3892/ijmm.2018.3996 30483753 PMC6317688

[241] Yan M Chen C Gong W Yin Z Zhou L Chaugai S miR-21-3p regulates cardiac hypertrophic response by targeting histone deacetylase-8. *Cardiovasc Res.* (2015) 105:340–52. 10.1093/cvr/cvu254 25504627

[242] Wang F Fang Q Chen C Zhou L Li H Yin Z Recombinant adeno-associated virus-mediated delivery of MicroRNA-21-3p lowers hypertension. *Mol Ther Nucleic Acids.* (2018) 11:354–66. 10.1016/j.omtn.2017.11.007 29858071 PMC5992325

[243] Li Y Deng S Peng J Wang X Essandoh K Mu X MicroRNA-223 is essential for maintaining functional β-cell mass during diabetes through inhibiting both FOXO1 and SOX6 pathways. *J Biol Chem.* (2019) 294:10438–48. 10.1074/jbc.RA119.007755 31118273 PMC6615686

[244] Lu H Buchan RJ Cook SA. MicroRNA-223 regulates Glut4 expression and cardiomyocyte glucose metabolism. *Cardiovasc Res.* (2010) 86:410–20. 10.1093/cvr/cvq010 20080987

[245] Suresh Babu S Thandavarayan RA Joladarashi D Jeyabal P Krishnamurthy S Bhimaraj A MicroRNA-126 overexpression rescues diabetes-induced impairment in efferocytosis of apoptotic cardiomyocytes. *Sci Rep.* (2016) 6:36207. 10.1038/srep36207 27827458 PMC5101812

[246] Wang D Wang H Liu C Mu X Cheng S. Hyperglycemia inhibition of endothelial miR-140-3p mediates angiogenic dysfunction in diabetes mellitus. *J Diabetes Complications.* (2019) 33:374–82. 10.1016/j.jdiacomp.2019.02.001 30862410

[247] Jordan SD Krüger M Willmes DM Redemann N Wunderlich FT Brönneke HS Obesity-induced overexpression of miRNA-143 inhibits insulin-stimulated AKT activation and impairs glucose metabolism. *Nat Cell Biol.* (2011) 13:434–46. 10.1038/ncb2211 21441927

[248] Wyss CB Duffey N Peyvandi S Barras D Martinez Usatorre A Coquoz O Gain of HIF1 Activity and Loss of miRNA let-7d promote breast cancer metastasis to the brain via the PDGF/PDGFR Axis. *Cancer Res.* (2021) 81:594–605. 10.1158/0008-5472.CAN-19-3560 33526470

[249] Ward JA Esa N Pidikiti R Freedman JE Keaney JF Tanriverdi K Circulating cell and plasma microRNA profiles differ between non-ST-Segment and ST-Segment-Elevation myocardial infarction. *Fam Med Med Sci Res.* (2013) 2:108. 10.4172/2327-4972.1000108 24432306 PMC3890357

[250] Goren Y Meiri E Hogan C Mitchell H Lebanony D Salman N Relation of reduced expression of MiR-150 in platelets to atrial fibrillation in patients with chronic systolic heart failure. *Am J Cardiol.* (2014) 113:976–81. 10.1016/j.amjcard.2013.11.060 24462065

